# Discovery of SARS-CoV-2 antiviral synergy between remdesivir and approved drugs in human lung cells

**DOI:** 10.1038/s41598-022-21034-5

**Published:** 2022-11-02

**Authors:** Xammy Nguyenla, Eddie Wehri, Erik Van Dis, Scott B. Biering, Livia H. Yamashiro, Chi Zhu, Julien Stroumza, Claire Dugast-Darzacq, Thomas G. W. Graham, Xuanting Wang, Steffen Jockusch, Chuanjuan Tao, Minchen Chien, Wei Xie, Dinshaw J. Patel, Cindy Meyer, Aitor Garzia, Thomas Tuschl, James J. Russo, Jingyue Ju, Anders M. Näär, Sarah Stanley, Julia Schaletzky

**Affiliations:** 1 Division of Infectious Diseases and Vaccinology, School of Public Health, University of California, Berkeley, CA 94720 USA; 2 The Henry Wheeler Center for Emerging and Neglected Diseases, 344 Li Ka Shing, Berkeley, CA 94720 USA; 3 Department of Molecular and Cell Biology, Division of Immunology and Pathogenesis, University of California, Berkeley, CA 94720 USA; 4 Department of Nutritional Sciences & Toxicology, University of California, Berkeley, CA 94720 USA; 5 Innovative Genomics Institute, University of California, Berkeley, CA 94720 USA; 6 Center for Genome Technology and Biomolecular Engineering, Columbia University, New York, NY 10027 USA; 7 Department of Chemical Engineering, Columbia University, New York, NY 10027 USA; 8 Department of Chemistry, Columbia University, New York, NY 10027 USA; 9 Department of Molecular and Cell Biology, Division of Genetics, Genomics and Development, University of California, Berkeley, CA 94720 USA; 10 Laboratory of Structural Biology, Memorial Sloan-Kettering Cancer Center, New York, NY 10065 USA; 11 Laboratory of RNA Molecular Biology, Rockefeller University, New York, NY 10065 USA; 12 Department of Molecular Pharmacology and Therapeutics, Columbia University, New York, NY 10032 USA

**Keywords:** High-throughput screening, Mass spectrometry, Infectious diseases, Biochemistry, Drug discovery, Molecular medicine

## Abstract

SARS coronavirus 2 (SARS-CoV-2) has caused an ongoing global pandemic with significant mortality and morbidity. At this time, the only FDA-approved therapeutic for COVID-19 is remdesivir, a broad-spectrum antiviral nucleoside analog. Efficacy is only moderate, and improved treatment strategies are urgently needed. To accomplish this goal, we devised a strategy to identify compounds that act synergistically with remdesivir in preventing SARS-CoV-2 replication. We conducted combinatorial high-throughput screening in the presence of submaximal remdesivir concentrations, using a human lung epithelial cell line infected with a clinical isolate of SARS-CoV-2. This identified 20 approved drugs that act synergistically with remdesivir, many with favorable pharmacokinetic and safety profiles. Strongest effects were observed with established antivirals, Hepatitis C virus nonstructural protein 5A (HCV NS5A) inhibitors velpatasvir and elbasvir. Combination with their partner drugs sofosbuvir and grazoprevir further increased efficacy, increasing remdesivir’s apparent potency > 25-fold. We report that HCV NS5A inhibitors act on the SARS-CoV-2 exonuclease proofreader, providing a possible explanation for the synergy observed with nucleoside analog remdesivir. FDA-approved Hepatitis C therapeutics Epclusa® (velpatasvir/sofosbuvir) and Zepatier® (elbasvir/grazoprevir) could be further optimized to achieve potency and pharmacokinetic properties that support clinical evaluation in combination with remdesivir.

SARS-CoV-2, a positive-sense RNA betacoronavirus, is the causative pathogen for the novel coronavirus disease 2019 (COVID-19)^1^. SARS-CoV-2 infects human epithelial lung cells via interaction with the ACE2 receptor, followed by virus replication and spread^2^. Pneumonia and acute respiratory distress can be severe, with alveolar damage, blood clotting abnormalities, and unusual large-vessel strokes often weeks after infection^3,4^. Sequelae include impaired lung function due to pulmonary fibrosis^5^, myocardial and neurological events, and the need for a lung transplant^6–8^. With the appearance and expansion of new serious variants of SARS-CoV-2, there is a concern that the current vaccines may be less effective. In addition, the existing single-target therapeutics are prone to lose efficacy because of resistance, and numerous mutations in the target of Paxlovid, Main Protease (MPro), have already been documented^9^. There is therefore an urgent need to identify antivirals that, in combination with vaccine deployment, will rapidly contain the COVID-19 pandemic.

The sole FDA-approved treatment for COVID-19 is remdesivir (GS-5734), a broad-spectrum antiviral originally discovered to treat Hepatitis C virus (HCV) and Ebola^10–12^. Remdesivir is a 1′-cyano-substituted adenine C-nucleoside ribose analogue (Nuc), a prodrug that requires intracellular conversion to an active triphosphate metabolite (NTP), which interferes with the activity of viral RNA-dependent RNA-polymerases (RdRp)^13^. In non-human primate models of COVID-19, robust effects are seen^14,15^. However, in humans, the median recovery time in a phase III clinical trial treatment group was only reduced from 15 to 11 days^16^, while in other studies no significant improvement over standard of care was apparent^17,18^. As a prodrug given intravenously, remdesivir’s pharmacokinetic profile is highly complex with several active metabolites^15^. As remdesivir is not highly potent, diffusion-driven distribution to the target tissue seems to be limiting efficacy, prompting the evaluation of an inhaled formulation^19,20^. Alternative approaches to improve remdesivir efficacy are urgently needed.

In antiviral therapy, combination therapies are highly efficacious, safe, and less prone to resistance development^21,22^. Indeed, the combination therapies Epclusa® (velpatasvir/sofosbuvir) and Zepatier® (elbasvir/grazoprevir) have transformed Hepatitis C care^23^. Similar combination approaches for COVID-19 would be highly desirable, as they could increase potency of remdesivir and allow a vastly larger number of patients to be treated with a limited stockpile. Here, we report an unbiased high-throughput combinatorial screen to identify compounds that act synergistically with remdesivir in blocking SARS-CoV-2 induced cytopathic effect. From a library of 1200 FDA-approved compounds, we identified 20 compounds that show robust synergy with remdesivir. The largest effects are observed with HCV nonstructural protein 5 (NS5A)-targeting antivirals velpatasvir and elbasvir and with their commercial co-formulations Epclusa® (velpatasvir/sofosbuvir) and Zepatier® (elbasvir/grazoprevir). We report that both HCV NS5A inhibitors inhibit the exonuclease proofreader of SARS-CoV-2. This proofreader can remove nucleotide inhibitors incorporated into virus RNA during replication (such as remdesivir), reducing treatment efficacy^24^. The resulting ~ 25-fold increase in remdesivir potency is highly promising and identifies velpatasvir and elbasvir as excellent starting points for further optimization of SARS-CoV-2 exonuclease targeted therapeutics.

## Combinatorial high-throughput screen for compounds synergistic with remdesivir

**Figure 1:**
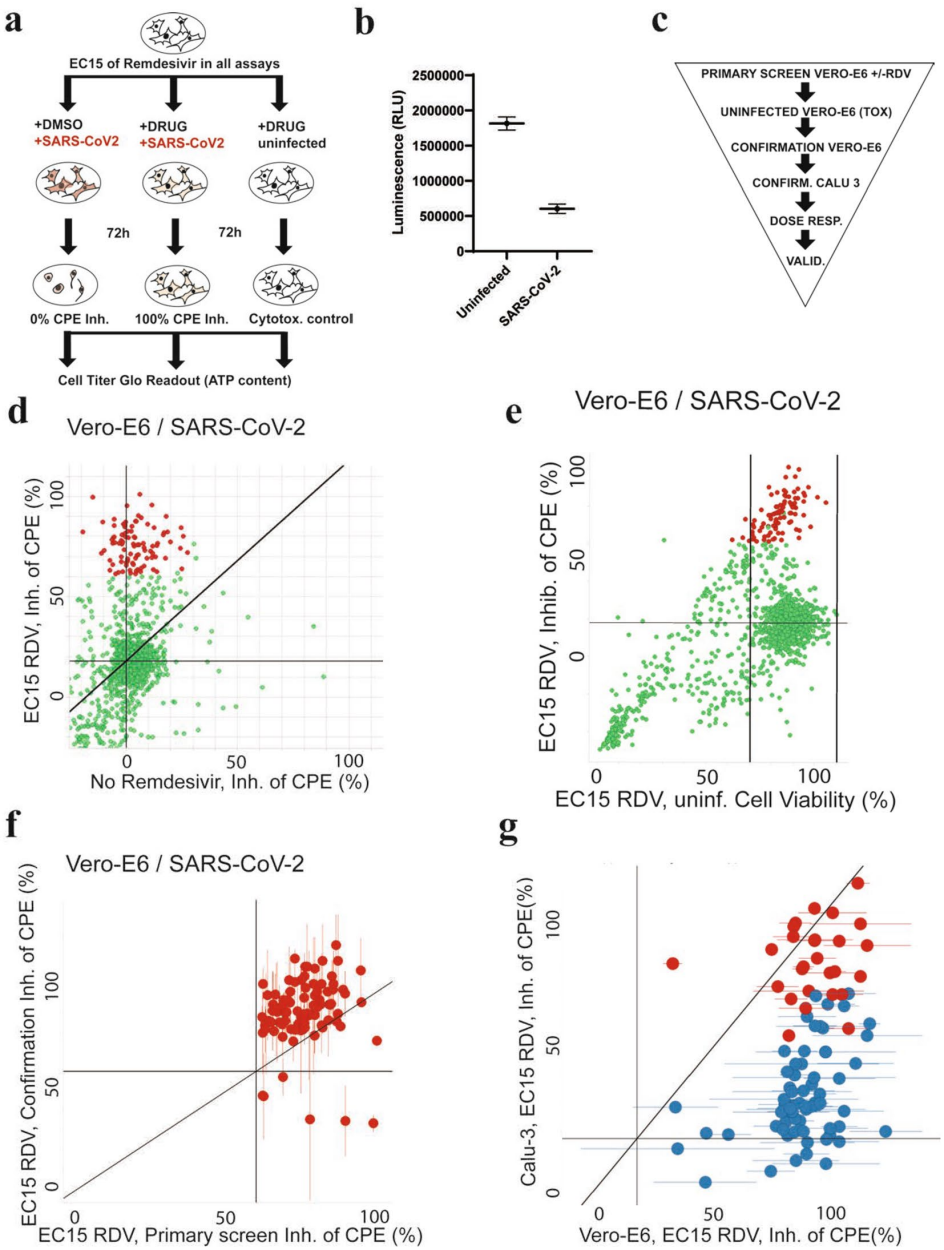
Primary screening results identifying compounds increasing antiviral effects of remdesivir (RDV). (**a**) Assay outline: Vero-E6 cells are added to 384 well plates, treated with DMSO (left panel) or drug (middle panel), infected with SARS-CoV-2 and incubated for 72 h to observe cytopathic effect (CPE; left panel). Effective drug treatment inhibits occurrence of CPE (middle panel). CPE is measured by quantifying ATP content in viable cells using a luminescent assay (Cell-Titer Glo). The right panel shows the cytotoxicity control, treating cells with drugs but without virus. (**b**) Screening assay performance. Average Luminescence is shown for the Vero E6 primary screen in presence of EC15 of remdesivir (n = 144, 24 wells each from 6 screening plates), error bars indicate standard deviation. “Uninfected”: positive control (equivalent to 100% inhibition of CPE), “SARS-CoV-2”: negative control, infected and treated with DMSO (equivalent to 0% inhibition of CPE). Z’ = 0.63 ± 0.04. (**c**) Screening paradigm outline in presence of EC15 of remdesivir. Cells infected with SARS-CoV-2 unless indicated. Dose resp.—Dose response; Valid.—Validation in orthogonal assays. **(d**) Primary screen results for 1200 approved drugs tested in Vero E6 cells infected with SARS-CoV-2 in the absence (*x*-axis) and presence (*y*-axis) of EC15 of remdesivir. Inhibition of CPE (%) is shown. The horizontal line indicates the background activity of EC15 of remdesivir (not subtracted). Diagonal line: 1:1 correlation. Red: high priority hits with a cutoff of > 60% inhibition of CPE in presence of remdesivir. (**e**) As in (**d**), but with cell viability data from cytotoxicity control (uninfected) on the *x*-axis. Vertical line: Cell viability of 70%. (**f**) Confirmation of > 95% of high priority hits from (**e**) after compound cherrypicking; assay conditions as in (**e**), Vero-E6 cells infected with SARS-CoV-2 in presence of EC15 of remdesivir; *x*-axis indicates primary screening results (Inhibition of CPE, %), *y*-axis confirmation results (Inhibition of CPE, %). Horizontal and vertical lines indicate hit progression cutoff from primary screen, diagonal line 1:1 correlation. Error bars indicate standard deviation. (**g**) 26 compounds (red; labeled) are active in both Vero E6 and human lung epithelial Calu-3 cells infected with SARS-CoV-2 in presence of EC15 of remdesivir. Inhibition of CPE (%) is shown on the *x*-axis for Vero E6, on the y-axis for Calu-3 cells.

We developed a robust high-throughput assay in SARS-CoV-2-infected monkey kidney epithelial Vero E6 and human lung epithelial Calu-3 cells (Fig. 1a–c)^25^, using a clinical isolate of SARS-CoV-2 virus (USA-WA1/2020)^26^. Vero E6 or Calu-3 cells were treated with a formulation of test compound at 40 μM + / − remdesivir at a previously titrated concentration causing 15% inhibition of virus-induced cytopathic effect (CPE, EC15), infected with SARS-CoV-2, and incubated for 72–96 h. We then measured CPE by quantifying ATP in viable cells using the luminescence-based Cell-Titer Glo assay. Of note, the EC15 concentration of remdesivir (0.3–1 μM) is comparable to the serum concentration of the main remdesivir metabolite in plasma (~ 0.4 μM)^14^, suggesting that the results of a screen performed under these conditions could be clinically meaningful. We also determined the average EC50 for remdesivir in our system to be 3 + / − 0.6 μM in Vero-E6 and 0.7 ± 0.1 μM in Calu-3 cells (Figure ED1), consistent with literature values (Vero-E6 EC50 0.6–11 μM, Calu-3 EC50 0.3–1.3 μM^27–29^).

With this assay, we conducted a primary screen of a library of ~ 1200 FDA-approved drugs with remdesivir in Vero E6 cells, achieving an average Z’ of 0.63 + / − 0.04 (Fig. 1b). A parallel screen in the absence of viral infection assessed compound toxicity. The primary screen identified 90 compounds with antiviral activity exclusively in the presence of EC15 remdesivir (Fig. 1d, red). None of the hit compounds showed significant toxicity in cells (Fig. 1e, red). More than 95% of hit compounds were confirmed in an additional Vero E6 assay (Fig. 1f). A secondary screen of these compounds was carried out in the Calu-3 assay; 28 of the initial hit compounds maintained strong antiviral activity in a background of EC15 of remdesivir across both cell lines (Fig. 1g, red).

**Figure 2:**
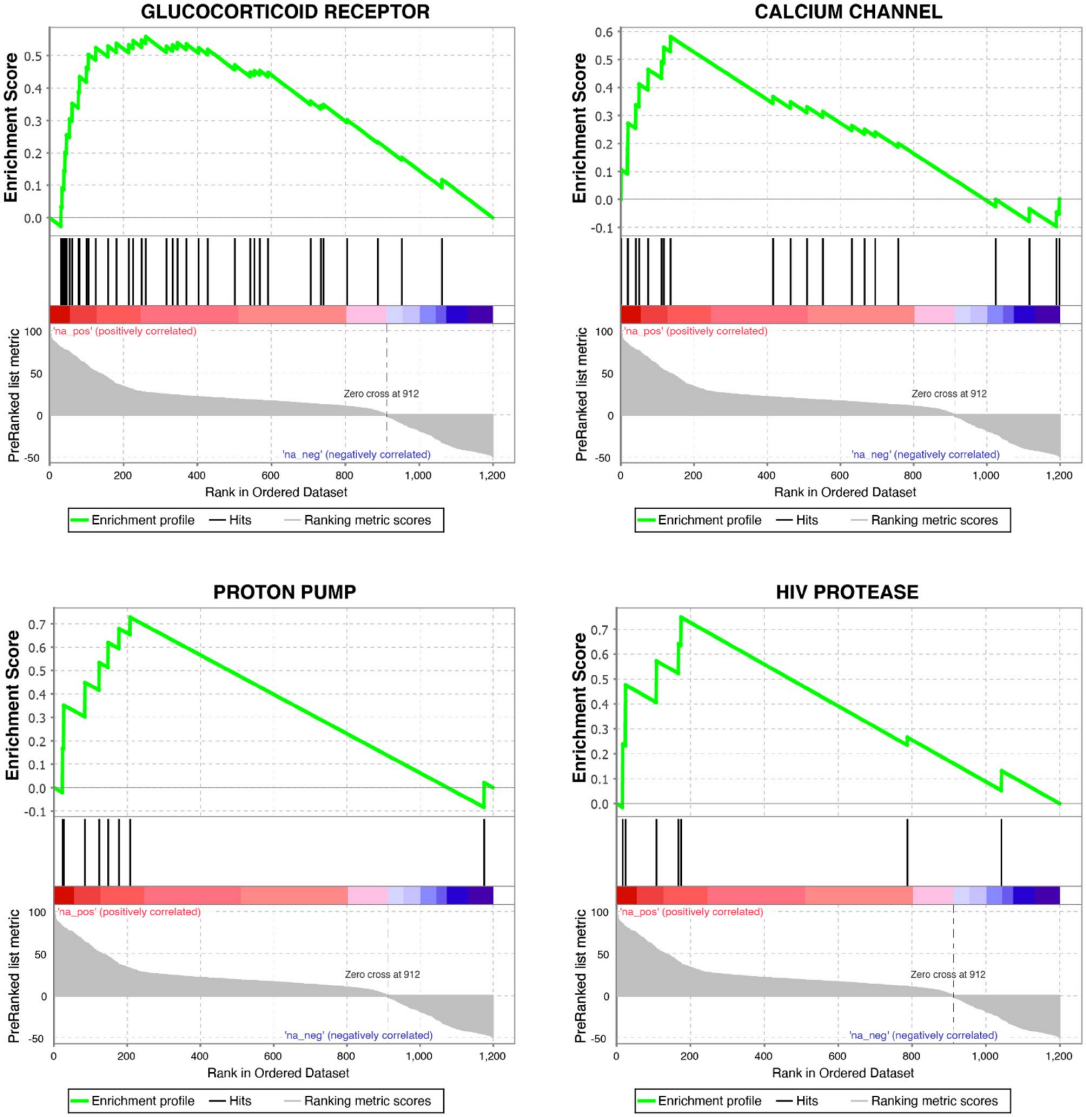
Gene set enrichment analysis of drug targets in combinatorial screen. GSEA enrichment plots provide the distribution of the enrichment score (green line) across compounds annotated to molecular targets (vertical black line), ranked in order of antiviral activity (left to right). The enrichment score (ES) reflects the degree to which a gene set is overrepresented at the top of a ranked list of compounds interacting with the given target. GSEA calculates the ES by walking down the ranked list of compounds interacting with the given target, increasing a running-sum statistic when a gene is in the gene set and decreasing it when it is not. Glucocorticoid receptor (*p* = 0.0001; FDR q value = 0.013), Calcium Channel (*p* = 0.004; FDR q value = 0.086), Proton pump (*p* = 0.003; FDR q value = 0.085) and HIV protease (*p* = 0.007; FDR q value = 0.095) are identified as targets enriched in the hitlist for the synergy screen in background of EC15 of remdesivir.

As all tested compounds are approved drugs annotated with their molecular targets, we conducted a gene set enrichment analysis to identify pathways preferentially targeted by hit compounds in the combinatorial screen (Fig. 2). We observed a statistically significant enrichment of compounds affecting the corticosteroid pathway (Fig. 2, GSEA *p* = 0.0001), as well as for calcium channel, proton pump and HIV protease modulation (Fig. 2, GSEA *p* = 0.004, 0.003), all of which have been implicated with antiviral effects in the literature^29–32^. Without remdesivir, none of the mentioned targets were enriched (*p* > 0.1, FDR q value > 0.36)^33^. This suggests that remdesivir makes SARS-CoV-2 uniquely vulnerable to inhibition of otherwise nonessential targets.

## Quantitation of synergistic effects with remdesivir in a dose response matrix

**Figure 3:**
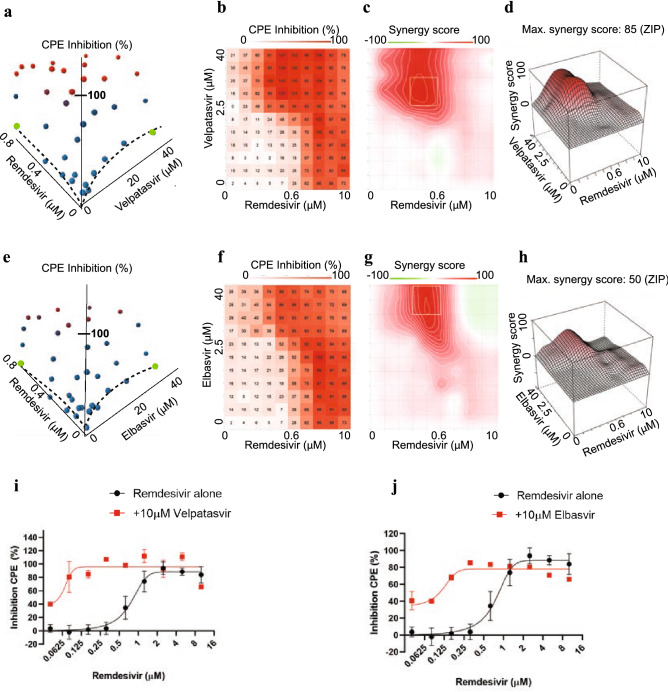
Synergy of direct-acting HCV antivirals velpatasvir (**a**)-(**d**) and elbasvir (**e**)-(**h**) with remdesivir in Calu-3 cells infected with SARS-CoV-2. (**a**) Three-dimensional plot showing synergy of combinations of velpatasvir (*x*-axis, up to 40 μM) and remdesivir (*y*-axis, up to 0.6 μM). *Z*-axis indicates CPE Inhibition (%). Marker colored using a gradient from blue (0% CPE inhibition) to red (100% CPE inhibition). Green—highest concentration of velpatasvir and remdesivir alone, reaching only ~ 20% Inhibition of CPE. Dashed line indicates dose response results of remdesivir and velpatasvir alone. (**b**) Two-dimensional representation of dose response interaction matrix. *X*-axis—Remdesivir (up to 10 μM), *y*-axis: Velpatasvir (up to 40 μM). Color gradient indicates Inhibition of CPE (%); white—0%, red—100%. (**c**) Topographic two-dimensional map of synergy scores determined in synergyfinder ^34^ from the data in (**a**) and (**b**), axes as in (**b**), color gradient indicates synergy score (red—highest score). (**d**) Three-dimensional surface plot representing synergy score (*z*-axis) for each compound combination. *X*-axis: remdesivir up to 10 μM, *y*-axis: velpatasvir up to 40 μM. (**e**), (**f**), (**g**), (**h**) as in (**a**), (**b**), (**c**), (**d**) but with elbasvir instead of velpatasvir. (**i**) Dose response of remdesivir alone (black) and in combination with 10 μM velpatasvir (red). (**j**) Dose response of remdesivir alone (black) and in combination with 10 μM elbasvir (red).

To identify the most promising drug combinations for use in the clinic, we conducted a dose–response interaction matrix analysis to quantitatively evaluate the synergy between screen hits and remdesivir. The matrix combined ten concentrations of remdesivir (up to 10 μM) with eleven concentrations of each screen hit (up to 40 μM), allowing us to test CPE in SARS-CoV-2-infected Calu-3 cells for 110 concentration combinations per remdesivir/compound pair. We then used computational zero interaction potency (ZIP) modeling to quantitatively determine if synergy was present^34^. The model combines both Loewe additivity and Bliss independence models, systematically assessing drug interaction patterns that may arise in a drug combination matrix. In this model, a value of < 0 signifies antagonism, 0–10 additive effects, and values > 10 show synergy between compound pairs. Strikingly, 20 compounds showed pronounced synergy with remdesivir in counteracting SARS-CoV-2-induced CPE, with maximal ZIP-scores of 29–87 (Fig. 3, Figure ED2): velpatasvir, elbasvir, dabrafenib, cilostazol, nimodipine, conivaptan hydrochloride, clobetasol, budesonide, drosiprenone, ezetimibe, ivosidenib, selexipag, meprednisone, nifedipine, omeprazole sulfide, quinapril, rifaximin, telmisartan, valdecoxib and zafirlukast.

Out of this list of candidates to combine with remdesivir, we prioritized velpatasvir, elbasvir, dabrafenib, cilostazol and nimodipine for detailed characterization based on the strength of the synergistic effect, mechanism of action, safety profile and the likelihood of clinical usefulness in context with best practices for COVID-19 treatment. Velpatasvir and elbasvir are hepatitis C antivirals targeting the HCV nonstructural protein 5 (NS5A), a replication co-factor with no clear homolog in SARS-CoV-2. Dabrafenib is a B-raf inhibitor used for melanoma chemotherapy, with an acceptable safety profile; B-raf inhibitors have been shown to have antiviral effects but have not been reported in the context of SARS-CoV-2^30,35^. Cilostazol is a widely prescribed, generically available PDE3 inhibitor, used to prevent stroke and treat intermittent claudication^36^. Nimodipine is a generically available calcium channel blocker used to treat hypertension with a favorable safety profile, acting on one of the druggable pathways enriched in the screen (Fig. 2)^37^.

The strongest candidate was velpatasvir (Fig. 3), with a maximum synergy score of 87. On its own, 40 μM velpatasvir inhibited SARS-CoV-2-induced CPE by less than 20%, as did remdesivir below 0.6 μM (Fig. 3a, dashed lines/green markers). In combination, 100% CPE inhibition was reached. This was also observed for combinations of submaximal concentrations (Fig. 3b). Statistically significant synergy was apparent for combinations from 1 μM of velpatasvir and 0.07 μM of remdesivir upwards, with a maximum being reached for combinations above 2.5 μM velpatasvir and 0.3 μM remdesivir (Fig. 3c, d). The presence of 10 μM velpatasvir shifts the EC50 for remdesivir from ~ 1 μM to 50 nM, a 20-fold difference (Fig. 3i, red). We identified another HCV NS5A inhibitor in the screen, elbasvir, which also demonstrated synergy in combination with remdesivir. Elbasvir increased inhibition of CPE exclusively when remdesivir was present (Fig. 3f), with a synergy score of 50 (Fig. 3g, 3h) and measurable effects at concentrations as low as 5 μM elbasvir and 0.2 μM remdesivir (Fig. 3f-h). In presence of 10 μM elbasvir, the EC50 for remdesivir was shifted > tenfold, from 0.7 μM to about 65 nM (Fig. 3j). Dabrafenib, cilostazol and nimodipine showed maximum synergy scores of 50, and close to 100% inhibition of CPE (Extended Data Figure ED3a, ED3b—dabrafenib, ED3c, ED3d—cilostazol, ED3e, ED3f.—nimodipine).

## Efficacy of clinically used coformulations

**Figure 4:**
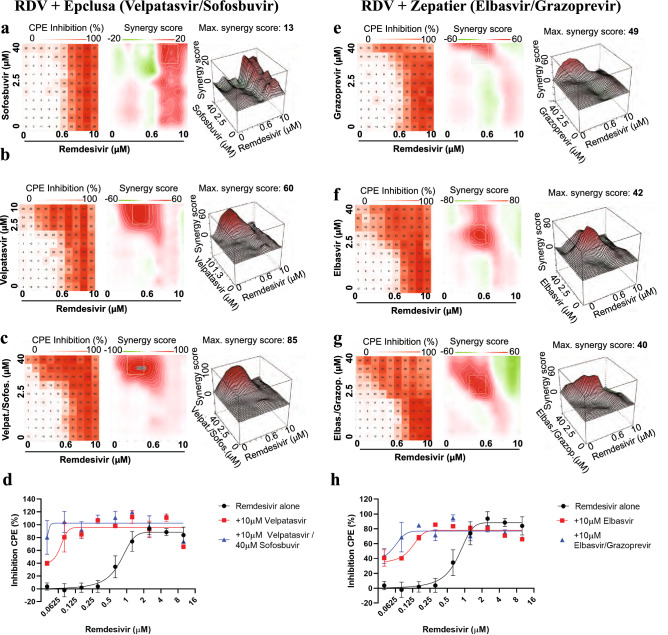
Velpatasvir and elbasvir enhance remdesivir activity when used in their commercially available co-formulation with sofosbuvir and grazoprevir. All experiments shown are in Calu-3 cells infected with SARS-CoV-2. (**a**) Left panel: Two-dimensional representation of dose response interaction matrix. *X*-axis—Remdesivir (up to 10 μM), *y*-axis: Sofosbuvir (up to 40 μM). Color gradient indicates Inhibition of CPE (%); white–0%, red-100%. Middle panel: Topographic two-dimensional map of synergy scores determined in synergyfinder ^34^ from the data in (**a**), axes as in (**b**), color gradient indicates synergy score (red – highest score). NB coloring scheme and *z*-axis autoscales to the highest value observed, inflating small changes for weak compounds such as sofosbuvir. (**b**) As in (**a**), but remdesivir combined with velpatasvir (up to 10 μM); (**c**) as in (**a**) but remdesivir combined with both velpatasvir (up to 10 μM) and sofosbuvir (up to 40 μM); axis indicates sofosbuvir concentration only, Velpatasvir is 4 × lower. **(d**) Dose response of remdesivir alone (black) and in combination with 10 μM velpatasvir (red) or 10 μM velpatasvir/40 uM sofosbuvir (blue). **(e**) as in (**a**), but remdesivir combined with grazoprevir (up to 40 μM). (**f**) as in (**a**), but remdesivir combined with elbasvir (up to 40 μM). (**g**) as in (**a**), but remdesivir combined with both elbasvir and grazoprevir (both up to 40 μM). (**h**) Dose response of remdesivir alone (black) and in combination with 10 μM elbasvir (red) or 10uM elbasvir/10 μM grazoprevir (blue).

As both velpatasvir and elbasvir are only available co-formulated with other antivirals, we tested the marketed drug combinations Epclusa® (velpatasvir 100 mg/sofosbuvir 400 mg, Gilead) and Zepatier® (elbasvir 50 mg/ grazoprevir 100 mg, Merck) in the dose response interaction matrix in SARS-CoV-2-infected Calu-3 cells. For Epclusa®, velpatasvir and sofosbuvir were added in 1:4 ratio as in their commercial coformulation. Sofosbuvir alone (up to 40 μM) showed very little synergistic effect with remdesivir (Fig. 4a), velpatasvir alone (up to 10 μM) reproduced the synergy observed previously (Fig. 4b), and the combination of velpatasvir/sofosbuvir (up to 10/40 μM, respectively) increased synergy with remdesivir further, enhancing activity of previously inactive remdesivir concentrations. The combination remdesivir/Epclusa® shifted the EC50 value of remdesivir ~ 25-fold, to 37 nM (Fig. 4c,d). For Zepatier®, the triple combination (elbasvir, grazoprevir, remdesivir) showed stronger synergy with remdesivir than elbasvir alone, shifting the EC50 value of remdesivir ~ 20-fold, to about 50 nM at 10 μM elbasvir/grazoprevir (Fig. 4g,h). Thus, commercially available drug combinations targeting HCV NS5A protein showed the strongest synergy with remdesivir in inhibiting SARS-CoV-2 to date.

## Orthogonal validation of prioritized antiviral drug combinations

**Figure 5:**
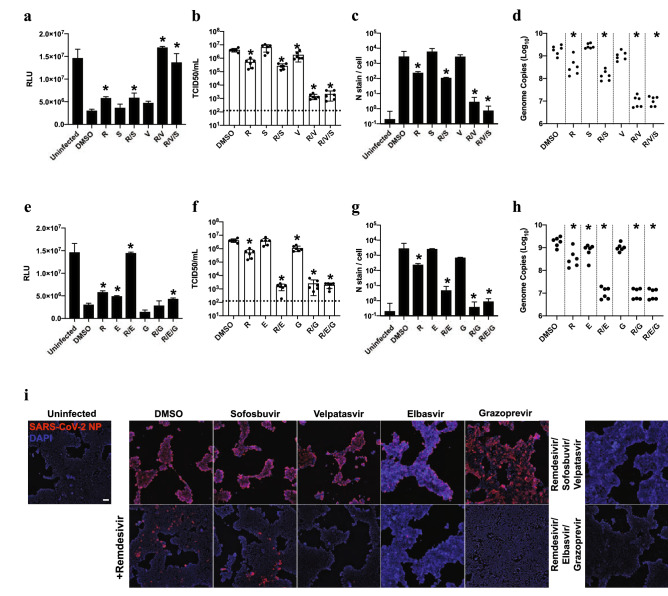
FDA-approved compounds synergize with low dose remdesivir to inhibit SARS-CoV-2 replication in orthogonal assays. (**a**) (**e**)Cell-titer Glo assay measuring ATP content of viable cells 96 h post-infection (hpi) in human lung epithelial Calu-3 cells infected with SARS-CoV-2 (MOI = 0.05). Drug was added at 40 μM except remdesivir, which was added at 0.625 μM (~ EC15), and velpatasvir, which was added at 10 μM to maintain the ratio of 1:4 in the coformulation with sofosbuvir. n = 3, error bars indicate Standard deviation. Asterisk indicates statistical significance with p < 0.05 relative to DMSO control. R: remdesivir; V: velpatasvir; S: sofosbuvir; E: elbasvir; G: grazoprevir (**b**) (**f**) infectious virus particle titer leading to 50% of cell death in Vero-E6 cells (TCID50) was determined from the supernatants of (**a**) and (**e**) 24hpi; other conditions as in (**a**) and (**e**). Dotted line indicates limit of detection in the assay. (**c**) (**g**) infection was quantified by direct visualization of virus particles by immunofluorescence assay, detecting number of SARS-CoV-2 nucleoprotein (N stain) 48hpi per infected cell. Other conditions were as in (**a**) and (**e**). For remdesivir/velpatasvir/sofosbuvir, results were not statistically significantly different from uninfected control cells (*p* = 0.18), as was the case with remdesivir/elbasvir/grazoprevir (*p* = 0.07) (**d**) (**h**) RT-qPCR quantifying SARS-CoV-2 genome equivalents of Calu-3 cells treated with the indicated drug combinations and infected with SARS-CoV-2 at MOI of 0.05 for 48hpi. (**i**) Representative images from (**c**) and (**g**), Calu-3 cells infected with SARS-CoV-2 48hpi. Scale bar corresponds to 100 μM.

We next assessed viral infectivity in a tissue culture infectious dose 50 (TCID50) assay, which determines the titer of infectious viral particles that leads to cell death in 50% of assayed wells. In this experiment, Calu-3 cells infected with SARS-CoV-2 were treated with EC15 remdesivir by itself or in combination with velpatasvir, sofosbuvir, elbasvir, grazoprevir, velpatasvir/sofosbuvir (Epclusa®), elbasvir/grazoprevir (Zepatier®), dabrafenib, cilostazol or nimodipine. The TCID50 assays (Fig. 5b,f) confirmed results seen in the CPE screening assay (Fig. 5a,e): on its own, remdesivir at its EC15 had only modest effects and velpatasvir or sofosbuvir had no significant effect; yet in combination, remdesivir/velpatasvir/sofosbuvir reduced the titer of infectious viral particles by ~ 1500-fold (Fig. 5b). Similar results were observed for elbasvir: the viral titer was reduced ~ 1500-fold in combination of elbasvir and remdesivir, with little to no effect from single agent treatment (Fig. 5f). Consistent with earlier results, the co-formulated combination of grazoprevir and elbasvir was synergistic with remdesivir (Fig. 5f; Fig. 4d). Dabrafenib, cilostazol and nimodipine also reduced viral titer in presence of remdesivir (Figure ED3h).

Similar results were obtained when we quantified infected cells by immunofluorescence microscopy. We treated Calu-3 cells with remdesivir and compound, infected cells with SARS-CoV-2, and stained for nuclei (DAPI) and SARS-CoV-2 nucleocapsid protein (N-protein, NP; Fig. 5c,g,i). While EC15 concentrations of remdesivir had little effect on viral replication, as indicated by distinct N-protein staining, its combination with velpatasvir, elbasvir, velpatasvir/sofosbuvir, elbasvir/grazoprevir, dabrafenib, cilostazol or nimodipine strongly reduced the number of infected cells (Fig. 5c,g,i and figure ED3h). In fact, cells treated with the commercial HCV antiviral combinations were statistically not significantly different from uninfected cells (Fig. 5c,g,i; RDV/velpatasvir/sofosbuvir *p* = 0.18, RDV/elbasvir/grazoprevir *p* = 0.07). Analyzing viral genome copy number from the supernatant of infected cells by RT-qPCR further confirmed the drastic effects of combining HCV antivirals with remdesivir in blocking SARS-CoV-2 replication (Fig. 5d,h and ED3j). We conclude that the approved HCV antiviral medications Epclusa® (velpatasvir/sofosbuvir) and Zepatier® (elbasvir/grazoprevir) are strongly synergistic with remdesivir in blocking SARS-CoV-2 replication, significantly reducing viral load of infected cells.

**Figure 6:**
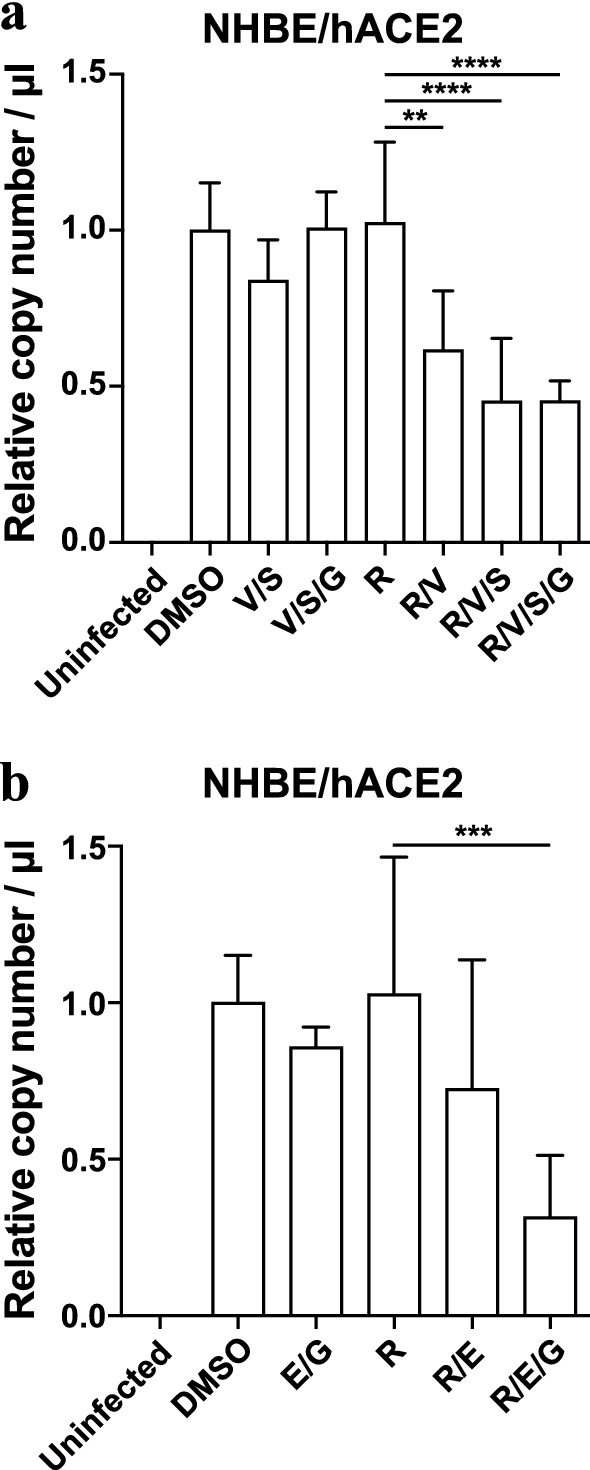
FDA-approved compounds synergize with low dose remdesivir in primary human bronchial epithelial cells. **(a) (b)** RT-qPCR quantifying SARS-CoV-2 genome equivalents at 96hpi of Normal Human Bronchial Epithelial (NHBE) cells transiently expressing hACE2 treated with the indicated drug combinations and infected with SARS-CoV-2 at MOI of 5. Drug was added at 40 μM except remdesivir, which was added at (**a**) 0.37 μM or (**b**) 0.13 μM, and velpatasvir, which was added at 10 mM to maintain the ratio of 1:4 in dosing with its combination sofosbuvir. Data represent four combined replicates from three independent experiments and genome copy number was normalized to DMSO within the same experiment. R: remdesivir; V: velpatasvir; S: sofosbuvir; E: elbasvir; G: grazoprevir.

To determine whether candidate compound antiviral synergy with remdesivir could be achieved in a more physiologically relevant cell type, we utilized primary normal human bronchial epithelial cells (NHBE) with transient overexpression of the human ACE2 receptor. As before, we found that treatment with remdesivir (EC15), velpatasvir, sofosbuvir, elbasvir, and grazoprevir as single agents, and in various clinically used combinations, possessed little effect on viral replication relative to the vehicle control conditions (Fig. 6a,b). In contrast, when the EC15 concentration of remdesivir was combined with the above compounds in various concentrations, we observed a significant synergistic effect against SARS-CoV-2 replication (Fig. 6a,b), consistent with the data in previous assays (Figs. 3,4,5). Again, the clinically used coformulations Epclusa® (Velpatasvir/Sofosbuvir) and Zepatier® (Elbasvir / grazoprevir) exhibited the strongest antiviral effect when combined with remdesivir (Fig. 6a,b). These data confirm the screening assay data in Calu-3 and Vero E6 cells and indicate antiviral synergy of HCV NS5A inhibitors with remdesivir can be observed in primary human lung cells infected with SARS-CoV-2.

## HCV NS5A inhibitors are inhibiting the SARS-CoV-2 exonuclease proofreader

Next, we studied mechanism of action, focusing on the strongest synergistic compound, velpatasvir. Its described HCV target NS5A is a part of the viral replication machinery, but has no known homolog in SARS-CoV-2 or other coronaviruses^42–45^. As velpatasvir on its own was not strongly active, we concluded that the potential target had to be non-essential in the absence of remdesivir, but had to become essential as remdesivir terminated RNA strands were generated. We speculated that the observed synergy effect could be explained by inhibition of the SARS-CoV-2 exonuclease proofreader.

**Figure 7:**
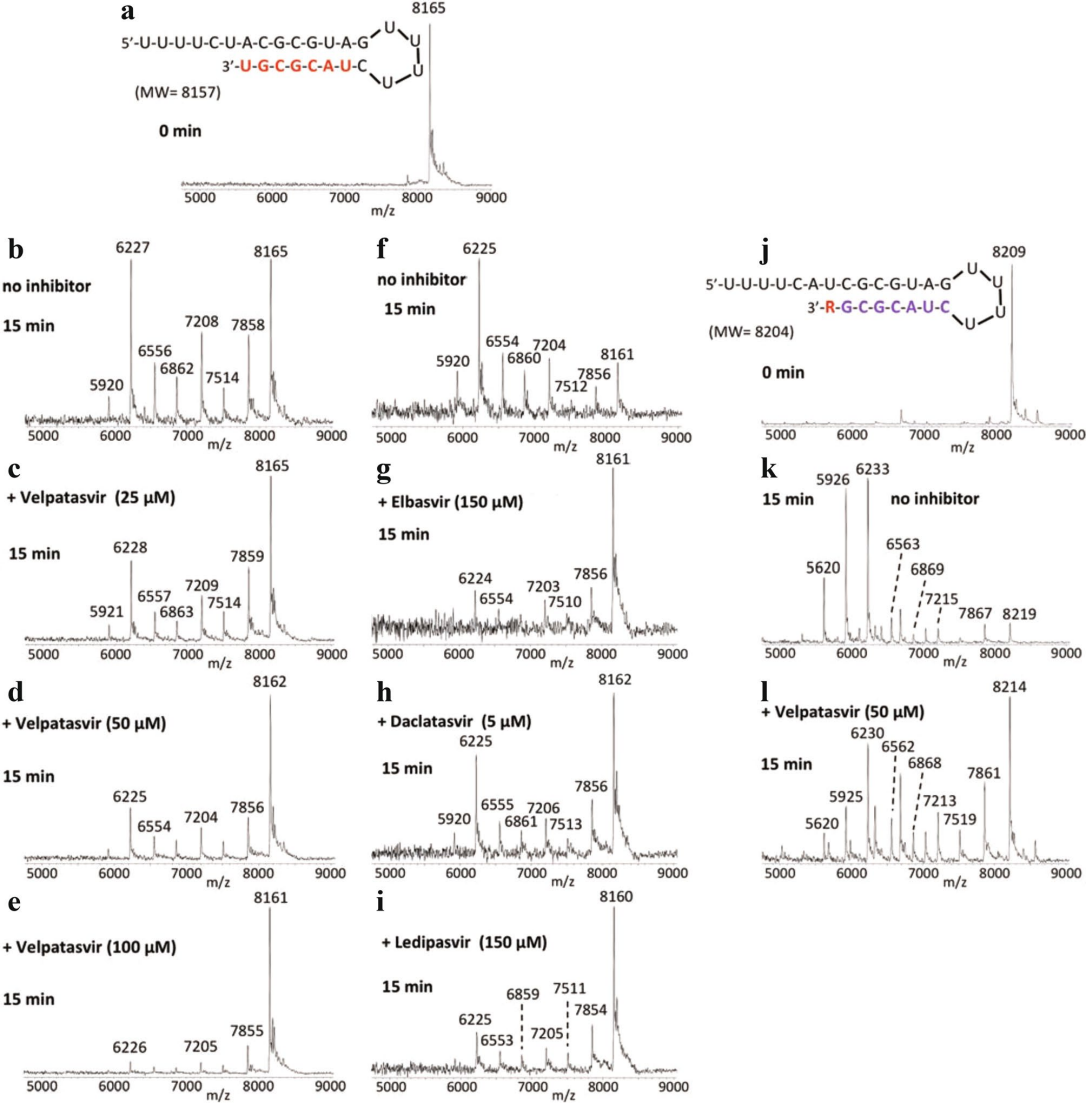
Inhibition of SARS-CoV-2 exonuclease activity by velpatasvir for remdesivir (R) terminated RNA. A mixture of 500 nM RNAs (sequences shown in (**a**), (**j**)) and 50 nM SARS-CoV-2 pre-assembled exonuclease complex (nsp14/nsp10) was incubated in buffer solution at 37 °C for 15 min in the absence (**b**), (**f**), (**k**) or presence of varying amounts of velpatasvir (**c-e**), (**l**), elbasvir (**g**), daclatasvir (**h**) or ledipasvir (**i**). The intact RNAs (**a**), (**j**) and the products of the exonuclease reaction (**b-i**), (**k-l**) were analyzed by MALDI-TOF MS. The signal intensity in each graph was normalized to the highest peak. In (**a-i**) the peak at 8165 Da corresponds to the full-length RNA and in (**j-l**) the peak at 8209 Da corresponds to remdesivir (R)-terminated RNA. In the absence of velpatasvir or elbasvir, exonuclease activity caused nucleotide cleavage from the 3’-end of the RNA as shown by the lower molecular weight fragments corresponding to cleavage of 1–7 nucleotides (**b**), (**f**), (**k**). In the presence of velpatasvir, elbasvir, daclatasvir or ledipasvir, exonuclease activity was significantly reduced as shown by the reduced intensities of the fragmentation peaks and increased peak of the intact RNA (**c-e**), (**g-i**), (**l**).

We used a mass spectrometry-based in vitro assay to investigate the exonuclease function of the SARS-CoV-2 nsp10 and nsp14 complex, which had been experimentally validated using a library of more than 25 known inhibitors for the SARS-CoV-2 polymerase complex^24,38–41^. RNA was incubated with pre-assembled SARS-CoV-2 exonuclease complex (nsp14/nsp10) in the absence or presence of varying amounts of velpatasvir. RNA and the cleavage products of the exonuclease reaction were analyzed by matrix-assisted laser desorption/ionization mass spectrometry (MALDI-TOF MS). The peak at 8165 Da corresponds to intact RNA (8157 Da expected, Fig. 7a). In the absence of velpatasvir, exonuclease activity caused nucleotide cleavage from the 3’-end of the RNA, as shown by the 7 lower molecular weight fragments corresponding to cleavage of 1–7 nucleotides, with only ~ 24% intact RNA remaining (Fig. 7b). Velpatasvir at 25, 50 and 100 μM reduced exonuclease activity in a concentration-dependent manner as shown by the reduced intensities of the fragmentation peaks and more prominent intact RNA peak (Fig. 7c-e). This was also observed with HCV NS5A inhibitor elbasvir, as well as other representatives of that drug class, daclatasvir and ledipasvir (Fig. 7g-i). These data indicate that HCV NS5A inhibitors can inhibit the SARS-CoV-2 exonuclease proofreader (nsp14/nsp10) complex, with velpatasvir demonstrating such activity in a dose-dependent manner.

Next, we determined the effect of velpatasvir on RNA terminated with the nucleotide analog remdesivir. Remdesivir (R)-terminated RNA was incubated with the pre-assembled nsp14/nsp10 complex in the absence or presence of 50 µM velpatasvir. The peak at 8209 Da represent intact R-terminated RNA (expected 8204, Fig. 7j). MALDI-TOF MS analysis demonstrated that in the absence of velpatasvir, exonuclease activity caused cleavage of 1–8 nucleotides from the 3’-end of the RNA as shown by the lower molecular weight fragments (Fig. 7k). When 50 µM velpatasvir was added, exonuclease activity was inhibited as shown by the reduced intensities of the fragmentation peaks and increased peak of the intact RNAs (Fig. 7l). Thus, the SARS-CoV-2 exonuclease activity is substantially inhibited by velpatasvir for both uracil-terminated and remdesivir-terminated RNA, offering a possible explanation for the synergistic effects of HCV NS5A inhibitors and remdesivir in Calu-3 and primary human lung cells infected with SARS-CoV-2.

## Discussion

Combination therapy cures Hepatitis C and enables long-term HIV suppression without significant development of resistance. Such therapy is highly desirable for the SARS-CoV-2 pandemic, but typically takes more than 10 years to develop. Here, we identify 20 FDA-approved compounds that have potential to improve efficacy of remdesivir and could be further optimized to make them clinically useful, significantly shortening timelines compared to de novo drug discovery.

Most strikingly, the drug class of HCV NS5A-inhibitors showed strong synergy with remdesivir in SARS-CoV-2 infected cells, both in immortalized and primary human lung cells, resulting in drastically reduced viral load. The molecular target of HCV NS5A inhibitors is a component of the HCV membrane-bound replication complex, with additional roles in virion assembly and modulation of host cell physiology, but no clear homolog in SARS-CoV-2^42–45^. Our data indicate that HCV NS5A inhibitors inhibit the SARS-CoV-2 exonuclease proofreader. This could explain why NS5A inhibitors by themselves do not act as strong antivirals in cell culture^29^, where virus replication is optimal and the proofreader might not be essential; under conditions where virus replication is severely impaired—in the presence of nucleoside inhibitors such as remdesivir—the proofreader could become essential, demonstrating the principle of synthetic lethality. Importantly, this class of molecules—despite being sought after—have remained elusive;  they do not show activity when tested on their own in direct antiviral screening approaches. Only recently, molecular docking combined with biochemical, in vitro assays for Nsp14/10 activity have yielded exonuclease proofreader inhibitors^46^. Importantly, these less potent compounds also do show some synergy with remdesivir, providing independent evidence that inhibition of the proofreader can be synergistic with RdRp inhibition in cells. Indeed, the exonuclease proofreader of coronaviruses is highly divergent from human exonucleases while being strongly conserved within coronaviruses, making it an ideal antiviral target with potential for pan-coronavirus use^46^. Our unbiased combinatorial approach has identified new classes of potentially useful compounds that would have remained obscure in single-agent screening approaches.

Given the promising in vitro data in the current study, it is important to consider the potency and pharmacokinetics of these drugs to understand their potential to be used in the clinic. A critical question to consider is if the concentrations of remdesivir and HCV NS5A inhibitors required for antiviral synergy in our in vitro systems are achievable in patients. The intracellular concentration of the active remdesivir metabolite in the human lung is estimated to be between 4–10 μM, close to its 7 μM IC50 and well below the 18 μM IC90 that would be needed to fully inhibit the virus^19^. Due to systemic toxicity, remdesivir cannot be dosed higher^19^; recent inhalation trials aim to increase lung concentration by changing route of administration. The 25-fold shift in potency reported in this study could move the IC90 from 18 μM to ~ 0.7 μM in the example above, well below the estimated intracellular concentration of 4–10 μM, putting more robust virus eradication within reach. However, the μM potency of velpatasvir and other NS5A inhibitors is likely too low to allow direct use in patients, in light of their nM exposure after oral administration^47^; this is not unexpected for repurposing approaches, where the new target is not the one the drug was initially designed for. However, different avenues are possible: higher doses in preclinical animal models could be investigated to increase exposure, supported by an extremely low toxicity of velpatasvir in rodents (NOAEL > 1500 mg/kg/day in mice, no clinical signs observed)^47^. Different routes of administration such as inhalation and specialized formulations such as nanoparticles could be tried^48,49^. The identified compounds are excellent starting points with favorable toxicity profiles for lead optimization studies; many analogs have been generated during preclinical development of HCV NS5A inhibitors and should be tested in the reported assays; structure–activity-relationship knowledge already exists from numerous HCV optimization campaigns. While some optimization will be needed, the lead optimization process is likely significantly shortened relative to de novo drug discovery.

In addition to HCV antivirals, we found 18 more synergistic combinations between remdesivir and approved drugs with favorable safety profiles and a wide range of pharmacokinetic properties that could be studied further and evaluated for their therapeutic usefulness, including dabrafenib, nimodipine and cilostazol. We also identified the well-tolerated and widely used steroids budesonide and meprednisone as showing robust synergy with remdesivir, supporting the notion that steroids can have direct antiviral effects (Figure ED2)^50,51^. These findings open up the possibility to find dual-action steroid-remdesivir combinations that have antiviral effects early in infection and exert immunomodulatory effects, as achieved with dexamethasone, later. Synergy with remdesivir was also observed for compounds modulating calcium channel and proton pump activity, consistent with well-established modulation and exploitation of host cell calcium signaling during infection^31,32,52^. We identified as synergistic with remdesivir the generic calcium-channel blockers nimodipine and nifedipine which are widely used as anti-hypertensives and have excellent safety profiles^37^. Omeprazole sulfide is a metabolite of omeprazole (Prilosec), an over-the-counter proton pump inhibitor to treat reflux, that has been previously identified as enhancing the effect of remdesivir on SARS-CoV-2^53^. Interestingly, several proton pump inhibitors have been strong hits in other SARS-CoV-2 repurposing screening campaigns^54^.

It is important to note that synergistic effects in cells could also be due to pharmacokinetic interactions. For example, NS5A inhibitors can inhibit the membrane transporters P-gp, BCRP, OATP1B1 and OATP1B3^55^. Those transporters reduce intracellular drug concentrations, and their inhibition could increase the apparent potency of remdesivir. However, analysis of known transporter inhibitors in our compound collection reveals only modest enrichment of OATP1B1 inhibitors, and no enrichment for P-gp, BCRP, and OATP1B3 inhibitors (Figure ED4). This suggests that the observed synergy is not likely due to pharmacokinetic interactions only.

Taken together, our study leverages unbiased combinatorial screening to discover compounds synergistic with antiviral remdesivir. We identify 20 promising combinations between remdesivir and approved drugs with a favorable safety profile and a wide range of pharmacokinetic properties. Among these, combining remdesivir with the HCV NS5A inhibitor combinations Epclusa (velpatasvir/sofosbuvir) and Zepatier (elbasvir/grazoprevir) increased remdesivir potency 25-fold and practically eliminated SARS-CoV-2 from infected cells, including from primary human lung cells, to our knowledge the strongest synergy with remdesivir observed to date. Synergistic combinations are synthetically lethal and strong candidates for further studies in animal models of SARS-CoV-2 infection and optimization, including administration and formulation, for possible clinical evaluation in COVID-19 patients. We provide evidence that HCV NS5A compounds such as velpatasvir and elbasvir are SARS-CoV-2 exonuclease proofreader inhibitors, which provides an intriguing explanation for the observed synthetic lethality with RdRp inhibition through remdesivir, and identifies a class of molecules that has been sought after but largely remained elusive through direct antiviral screening approaches. Exonuclease proofreader inhibitors would be highly desirable in the clinic for potential combination treatment with nucleotide analogs and could improve efficacy, prevent the development of resistance and allow the addition of more therapeutic options as vaccine protection begins to wane. Indeed, encouraging clinical data has recently been reported for Epclusa® in mild to moderate COVID-19^56^. Hepatitis C NS5A therapeutics Epclusa® (velpatasvir/sofosbuvir) and Zepatier® (elbasvir/grazoprevir) would be excellent starting points for optimization of potency and pharmacokinetic properties to enable clinical evaluation in COVID-19 patients in combination with remdesivir.

## Methods

### Cells and virus

Vero E6 and Calu-3 cells (Calu-3:ATCC HTB-55; Vero E6: ATCC, CRL-1586) were maintained in high glucose DMEM (Gibco, Waltham, MA, USA) supplemented with 10% FBS (R&D Systems, Minneapolis, MN, USA), 1X GlutaMAX (Gibco, Waltham, MA, USA), and 1X PenStrep (Gibco, Waltham, MA, USA) at 37 °C and 5% CO_2_. Normal Human Bronchial Epithelial (NHBE) cells transiently expressing hACE2 were obtained from Lonza (Basel, Switzerland) (cat. no. CC-2540) and maintained in bronchial epithelial cell growth medium (Lonza, Basel, Switzerland; CC-3170). To generate a master viral stock, Vero E6 were plated in T175 flasks (Nunc, Roskilde, Denmark) and allowed to grow to ~ 80% confluency before infection with the USA-WA1/2020 strain of SARS-CoV-2 (BEI Resources, Manassas, VA; NR-52281). At 72hpi, dramatic CPE was observed and the flasks were freeze-lysed at − 80°C. After thaw, lysate was collected and centrifuged at 3000 × rpm for 20 min to pellet cell debris (Beckman Coulter Allegra X-14R). This procedure was repeated for a second passage working stock with collection at 48hpi and titered by TCID50 assay.

### Compound preparation and drug screening

The FDA-approved drug library containing 1200 small molecule compounds (TargetMol, Wellesley Hills, MA, L4200) was stored at 10 mM in dimethyl sulfoxide (DMSO) in 384-well master plates. Resupply for velpatasvir, elbasvir, daclatasvir and ledipasvir was also obtained from TargetMol. Remdesivir was stored at 10 mM in DMSO (T7766, TargetMol). 2500 Vero E6 (12 μl/well) or 10,000 Calu-3 (12 μl/well) were seeded in 384-well white optical-bottom tissue culture plates (Nunc) with the Multidrop Combi liquid handling instrument (Thermo Fisher Scientific, Waltham, MA). Cells were allowed to adhere and expand, 24 h for Vero E6 and 48 h for Calu-3, at 37 °C and 5% CO_2_. For the primary screen, confirmation and synergy dose response interaction matrix analysis, compounds were prediluted to 8 × final concentration in high glucose DMEM. 3 μl compound was transferred from dilution plates using a Cybio Well vario liquid handler (Analytik Jena, Jena, Germany) to cells, leading to a final concentration of DMSO at 0.44% in the assay plate (v/v). Primary screen and confirmation was performed at 40 μM compound, dose responses were generated by 2 × dilutions starting at 40 μM or 10 μM. For synergy experiments, EC15(± 5) of remdesivir was empirically determined and used for each experiment in combination with other drugs as indicated above. Final DMSO was maintained at 0.44%—0.8% (v/v). Cells were incubated at 37 °C and 5% CO_2_ for 1 h before infection. Viral inoculum was prepared such that the final MOI = 0.05 upon addition of 6 μl/well viral inoculum. After complete CPE was observed in DMSO-treated, infected wells 72hpi for Vero-E6 and 96hpi for Calu 3, opaque stickers (Nunc) were applied to plate optical bottoms, and plates were developed with the CellTiter-Glo 2.0 reagent (Promega, Madison, WI) according to the manufacturer’s instructions. For Vero E6 reagent was diluted 1:1 (v/v) in PBS (Gibco, Waltham, MA, USA). Luminescence of developed plates was read on a Spectramax L (Molecular Devices, San Jose, CA). Each plate contained 24 wells uninfected/DMSO treated cells (100% CPE inhibition), and 24 wells infected/DMSO treated cells (0% CPE inhibition). Average values from those wells were used to normalize data and determine % CPE inhibition for each compound well. For duplicate plates, average values and standard deviations were determined. Z’ was determined as described^57^. Stastical significance was assessed using a two-tailed, heteroscedastic student’s t-test. Measurements were taken from distinct samples unless indicated otherwise. The data was plotted and analyzed with spotfire (Tibco) and GraphPad Prism. Synergy analysis was performed using synergyfinder, using a zero-interaction potency (ZIP) model^34^.

### GSEA analysis

Compounds were annotated with targets, pathways and mechanisms of actions using the Center for Emerging and Neglected Diseases’ database and for pharmacokinetic data and transporter inhibition data, the DrugBank database^58^. Each annotation property was tested for enrichment among the screening hits using the gene set enrichment analysis (GSEA) software as described^29,59,60^. The compounds annotated for each property were treated as part of the “gene set”. For each set of annotations, the background compound set was defined as the set of compounds annotated for any property. GSEA preranked analysis was performed using the compounds’ % CPE inhibition from each screen. Compound sets included in the analysis were between 5 and 500 compounds. Enrichment results with *p* < 0.01 and false discovery rate (FDR) q value < 0.1 were considered statistically significant. *P* values were generated using a one-sided hypergeometric test^61^.

### Immunofluorescence microscopy analysis (IFA) and RT-qPCR

50,000 Calu-3 cells (50 μl /well) were seeded in 96-well black optical-bottom tissue culture plates (Nunc). 24 h post-seeding, drug combinations were added to the cells in 25 μl DMEM and incubated at 37 °C and 5% CO_2_ for 1 h before infection. 25 μl viral inoculum was added for MOI = 0.05. At 48hpi, 75 μl supernatant was collected for RT-qPCR analysis. Cells were then washed with PBS, fixed in 4% paraformaldehyde (PFA) in PBS for 15 min, washed again with PBS, permeabilized with 0.2% saponin in blocking buffer (2% BSA, 2% FBS in PBS) for 30 min at room temperature (RT), and incubated with 1:1000 mouse antibody specific for SARS-CoV-2 nucleocapsid protein (Sino Biological, Beijing, China 40,143-MM05) overnight at 4 °C in blocking buffer consisting of 2% FBS and 2% BSA. The following day, plates were washed 3X with PBS, incubated with a 1:1000 dilution of goat anti-mouse AlexaFluor647 antibody (Abcam, Cambridge, United Kingdom) and DAPI/Hoechst (Invitrogen) in blocking buffer for 1 h at RT, washed again 3 × with PBS, fixed in 4% PFA, and replaced in PBS. Plates were fluorescently imaged using an Image Xpress Micro 4 (Molecular Devices). Images were analyzed for N stain per nuclei with the CellProfiler 3.1.9 software (Broad Institute, Cambridge, MA).

For RT-qPCR, 75 μl supernatants were collected at 48hpi and inactivated 1:1 in 1X DNA/RNA Shield for RNA extraction and RT-qPCR analysis (Zymo Research, Irvine, CA). RNA was extracted using the QIAamp Viral RNA Mini Kit (Qiagen, Hilden, Germany) according to the manufacturer's instructions. In brief, 140 μl of each sample was mixed with 560 μl of carrier RNA-containing AVL and incubated for 10 min at RT. After addition of 560 μl of 100% ethanol, the samples were spun through columns. The columns were washed sequentially with 500 μl of AW1 and 500 μl AW2, and RNA was eluted using 50 μl of RNase-free water. RT-qPCR reactions with TaqPath master mix (Thermo Fisher) were assembled following the manufacturer’s instructions.

For a 10 µl reaction, 2.5 µl of 4 × TaqPath master mix was combined with 0.75 µl of SARS-CoV-2 (2019-nCoV) CDC N1 qPCR Probe mixture (Integrated DNA Technologies, Cat. #10,006,606, Primer sequences: 2019-nCoV_N1-F: GAC CCC AAA ATC AGC GAA AT; 2019-nCoV_N1-R: TCT GGT TAC TGC CAG TTG AAT CTG; 2019-nCoV_N1-P: FAM-ACC CCG CAT TAC GTT TGG TGG ACC-BHQ1), 3 µl RNA sample, and 3.75 µl water. RT-qPCR was performed on a BioRad CFX96 instrument with the following cycle: (1) 25 °C for 1 min, (2) 50 °C for 15 min, (3) 95 °C for 2 min, (4) 95 °C for 3 s, (5) 55 °C for 30 s (read fluorescence), (6) go to step 4 for 44 repetitions. Quantification cycle (Cq) values were determined using the second derivative peak method^62^. Custom code written in MATLAB (available at https://gitlab.com/tjian-darzacq-lab/second-derivative-cq-analysis) was used to take the numerical second derivative of fluorescence intensity with respect to cycle number, using a sliding window of ± 3 cycles. The peak of the second derivative was fit to a parabola, whose center was taken to be the Cq value^62^.

### 96 well CellTiter-Glo 2.0 and TCID50 assay

40,000 Calu-3 cells (50 µl/well) were seeded in 96-well white optical-bottom tissue culture plates (Nunc). 48 h post-seeding, drug combinations were added to the cells in 25 µl DMEM and incubated at 37 °C and 5% CO_2_ for 1 h before infection. 25 µl viral inoculum was added for MOI = 0.05. At 24hpi, 25 µl supernatant was saved for TCID50 assay. After complete CPE was observed in DMSO-treated, infected wells 96hpi, opaque stickers (Nunc) were applied to plate optical bottoms, and plates were developed with the CellTiter-Glo 2.0 reagent (Promega, Madison, WI), according to the manufacturer’s instructions. Luminescence of developed plates was read on a Spectramax L (Molecular Devices, San Jose, CA).

To quantify infectious particles secreted by cells in a TCID50 assay, 25 μl of supernatant from infected, combination-treated cells was collected at 24hpi/drug treatment and tenfold serially diluted in DMEM. Each dilution was applied directly to eight wells in 96-well plates (Corning) pre-prepared with Vero E6 cells, then incubated for three days at 37 °C and 5% CO_2_. TCID50/mL for each sample was calculated by determining the dilution factor required to produce CPE, including syncytia formation, cell clearing and cell rounding, in half, or 4/8, of the wells. Limit of detection was determined as the concentration of virus resulting in CPE in 50% of the wells treated with the lowest dilution of sample.

### Reagents and purification of the SARS-CoV-2 exonuclease nsp14/nsp10 complex

For exonuclease experiments, remdesivir triphosphate (RDV-TP) was purchased from MedChemExpress (Monmouth Junction, NJ), sofosbuvir triphosphate (SOF-TP) was purchased from Sierra Bioresearch (Tucson, AZ), and UTP was purchased from Fisher Scientific. The RNA oligonucleotide (template-loop-primer) was purchased from Dharmacon (Horizon Discovery, Lafayette, CO). The 3′-exonuclease, referred to as nsp14, and its protein cofactor, nsp10, were cloned and expressed based on the SARS-CoV-2 genome sequence. The pRSFDuet-1 plasmids (Novagen) coding SARS-Cov-2 Nsp14 or nsp10 engineered with an N-terminal His-SUMO tag were prepared as follows: SARS-CoV-2 RNA isolated from the supernatant of SARS-CoV-2-infected Vero E6 cells was provided by Benjamin R. tenOever^63^. The sequence encoding nsp10 and nsp14 was reverse transcribed into cDNA using gene-specific primers and SuperScript III Reverse Transcriptase (ThermoFisher). nsp10 and nsp14 coding sequences were PCR amplified using forward and reverse gene specific primers flanked by BamHI and XhoI recombination sites, respectively. PCR products were digested with BamHI and XhoI and subsequently ligated into BamHI and XhoI-digested pRSFDuet-His6-sumo vector and sequence verified. pRSFDuet-His6-sumo is a modified pRSFDuet-1 vector (Novagen) bearing an N-terminal His6-SUMO-tag cleavable by the ubiquitin-like protease (ULP1).

NSP14 pRSF BamHI fw CGCGGATCC GCTGAAAATGTAACAGGACTCTTTAAA.

NSP14 pRSF XhoI rev CCCGCTCGAGCGG TCA CTGAAGTCTTGTAAAAGTGTTCCAGAGG.

RT primer NSP14 TTCTTGGCTATGTCAGTCATAGAACAAAC.

NSP10 pRSF BamHI fw CGCGGATCC GCTGGTAATGCAACAGAAGTGCCTGCC.

NSP10 pRSF XhoI_rev CCCGCTCGAGCGG TCA CTGAAGCATGGGTTCGCGGAGTTGATC.

RT_primer NSP10 GATGTTGATATGACATGGTCGTAACAGC.

The nsp14 and nsp10 proteins were expressed in Escherichia coli BL21-CodonPlus(DE3)-RIL (Stratagene). The bacteria were grown in Luria–Bertani medium supplemented with 50 mg/mL kanamycin at 37 °C to an OD600 of 0.6, induced with 0.4 mM isopropyl β-D-1-thiogalactopyranoside and 50 µM ZnCl_2_ overnight at 18 °C. Cells were collected via centrifugation at 5000 × g and equal volumes of the nsp14 and nsp10 bacterial cells were then mixed for nsp14-nsp10 protein complex purification. They were lysed via sonication in Lysis Buffer (500 mM NaCl, 20 mM imidazole, 20 mM Tris·HCl, pH 8.0, 1 mM phenylmethylsulfonyl fluoride). After centrifugation at 40,000 × g, the supernatant was loaded onto 5 mL Nickel Sepharose 6 fast flow resins (GE Healthcare) in a gravity flow column. The target protein was eluted using Lysis Buffer supplemented with 500 mM imidazole. The eluted protein was incubated with ULP1 (lab stock) during dialysis at 4 °C overnight against a buffer containing 20 mM Tris·HCl, pH 7.5, 20 mM imidazole, 150 mM NaCl, 100 µM ZnCl_2_, and 5 mM β-mercaptoethanol. Then the sample was loaded onto the HisTrap FF column (GE Healthcare) to remove His-SUMO tag, and the flow-through was collected. The target proteins were further purified through a Superdex200 10/300 gel filtration column (GE Healthcare) in a buffer containing 20 mM HEPES, pH 7.4, 150 mM NaCl, 1 mM MgCl_2_, and 1 mM dithiothreitol. The fractions corresponding to the nsp14 and nsp10 complex were detected by SDS-PAGE and collected. The protein sample was flash-frozen in liquid nitrogen and stored at − 80 °C.

### Extension reactions with SARS-CoV-2 RNA-dependent RNA polymerase to produce Remdesivir (RDV) terminated RNAs

10 µL of 10 µM RNA template-loop-primers (5’-UUUUCAUCGCGUAGUUUUCUACGCG-3’ for RDV-TP extension) in 1 × RdRp reaction buffer was annealed by heating to 75 °C for 3 min and cooling to room temperature. 5 µL of 8 µM RdRp complex (nsp12/nsp7/nsp8)^38^ in 1 × reaction buffer was added to the annealed RNA template-loop-primer solution and incubated for an additional 10 min at room temperature. Finally, 5 µL of a solution containing 0.2 mM RDV-TP in 1 × reaction buffer was added and incubation was carried out for 2 h at 30 °C. The final concentrations of reagents in the 20 µL extension reactions were 2 µM nsp12/nsp7/nsp8, 5 µM RNA template-loop-primer, and 50 µM RDV-TP. The 1 × reaction buffer contains the following reagents: 10 mM Tris–HCl pH 8, 10 mM KCl, 2 mM MgCl_2_ and 1 mM β-mercaptoethanol. Desalting of the reaction mixture was performed with an Oligo Clean & Concentrator kit (Zymo Research) resulting in ~ 10 µL purified aqueous RNA solutions. 1 µL of each solution was subjected to MALDI-TOF MS (Bruker ultrafleXtreme) analysis. The remaining ~ 9 µL extended template-loop-primer solutions were used to test exonuclease activity as described below.

### SARS-CoV-2 exonuclease reactions in the presence and absence of velpatasvir

The U-terminated RNA (Fig. 7), and the RDV extended RNA product from above (sequences shown in Fig. 7), were annealed by heating to 75 °C for 3 min and cooling to room temperature in 1 × exonuclease reaction buffer. To a 14 µL solution of 71.4 nM exonuclease complex (nsp14/nsp10) in 1 × exonuclease reaction buffer, 1 µL of DMSO with or without various concentrations of velpatasvir was added and incubated for 15 min at room temperature. Then 5 µL of the annealed RNA (2 µM) in 1 × exonuclease reaction buffer was added to the exonuclease/velpatasvir mixture and incubated at 37 °C for 15 min. The final concentrations of reagents in the 20 µL reactions were 50 nM nsp14/nsp10, 500 nM RNA, 0–100 µM velpatasvir and 5% DMSO. The 1 × exonuclease reaction buffer contains the following reagents: 40 mM Tris–HCl pH 8, 1.5 mM MgCl_2_ and 5 mM DDT. Following desalting using an Oligo Clean & Concentrator (Zymo Research), the samples were subjected to MALDI-TOF MS (Bruker ultrafleXtreme) analysis.

## Supplementary Information

Supplementary Information.

## Data Availability

The authors declare that the data supporting the findings of this study are available within the paper and its supplementary information files.

## Code availability

Custom code written in MATLAB for qRT-PCR analysis is available at https://gitlab.com/tjian-darzacq-lab/second-derivative-cq-analysis.
