# Supplementary material for: Discovery of SARS-CoV-2 antiviral synergy between remdesivir and approved drugs in human lung cells
Source: Sci Rep. 2022 Nov 2;12:18506. doi: 10.1038/s41598-022-21034-5 (PMC9628577; doi:10.1038/s41598-022-21034-5)
Supplement: Supplementary file 1 — Supplementary Information. [file 41598_2022_21034_MOESM1_ESM.pdf]

a

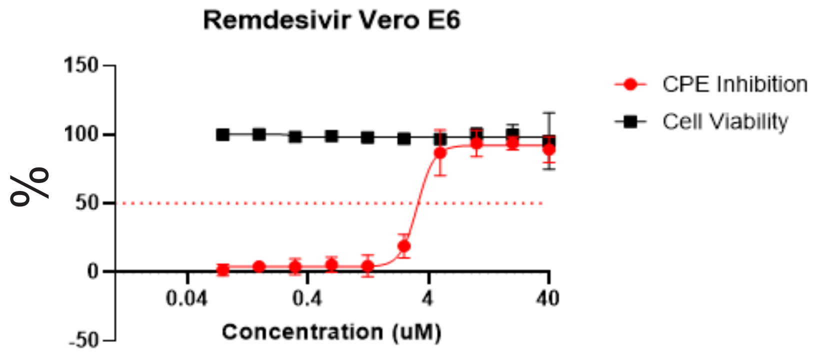

EC50= 3.08 uM +/- 0.62

b

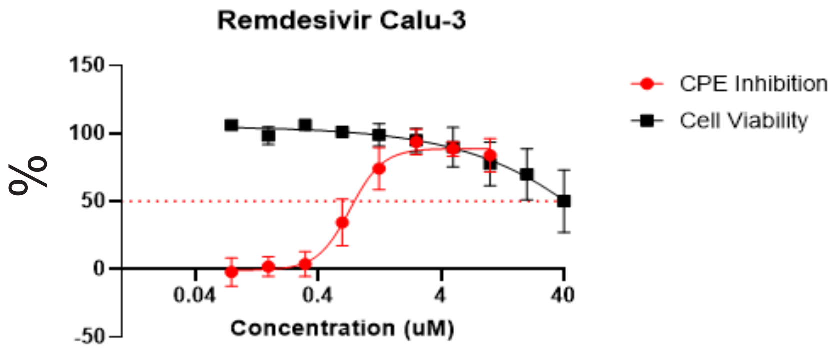

EC50= 0.69 uM +/- 0.10

Extended Data Figure 1. Remdesivir dose response curves averaged across all experiments in 384well assays in Vero E6 (a) and Calu-3 cells (b). Error bars indicate standard deviation. CPE inhibition (%) in SARS-CoV-2 infected cells in red, Cell viability (%) in uninfected cells in black. Data represent n=3 for Vero-E6 cells and n=4 for Calu-3 cells.

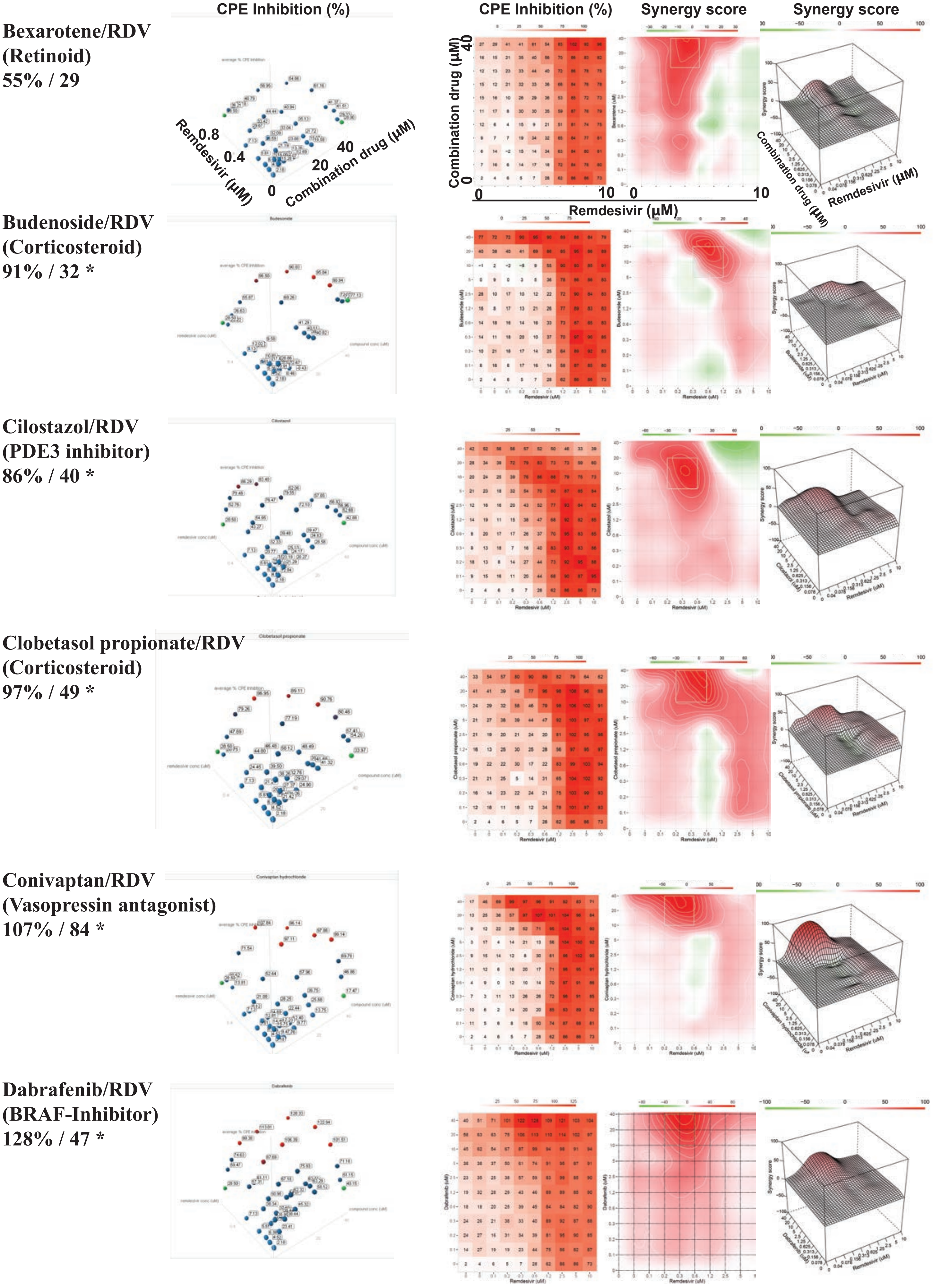

CPE Inhibition (%)
CPE Inhibition (%)
Synergy score
Synergy score
Bexarotene/RDV
(Retinoid)
55% / 29
Remdesivir (µM)
Combination drug (µM)
Remdesivir (µM)
Budenoside/RDV
(Corticosteroid)
91% / 32 *
Cilostazol/RDV
(PDE3 inhibitor)
86% / 40 *
Clobetasol propionate/RDV
(Corticosteroid)
97% / 49 *
Conivaptan/RDV
(Vasopressin antagonist)
107% / 84 *
Dabrafenib/RDV
(BRAF-Inhibitor)
128% / 47 *

Doxapram/RDV
(IV Analeptic, K channel inh.)
76% / 44

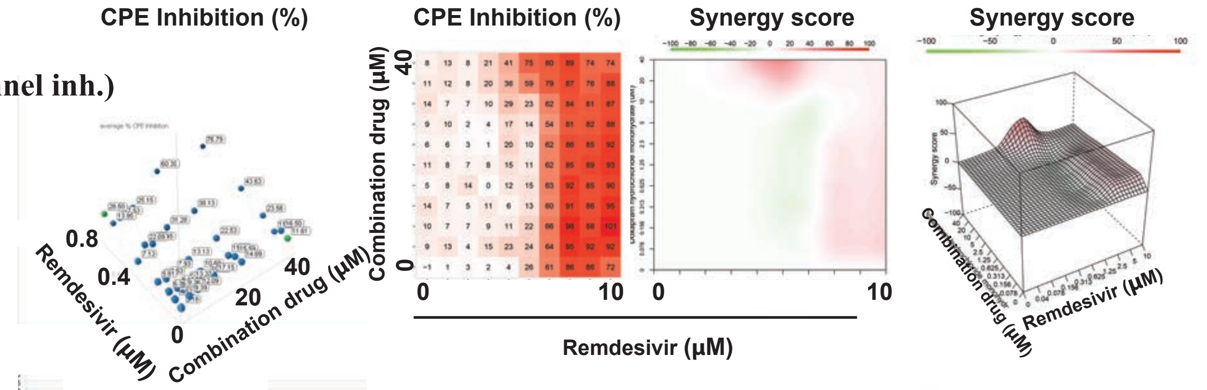

Drosiprenone/RDV
(Corticosteroid)
85 % / 29 *

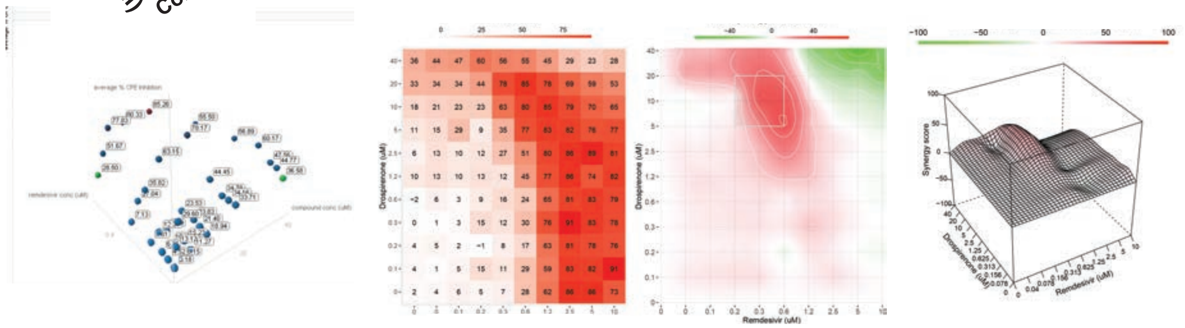

Elbasvir/RDV
(HCV NS5A inhibitor)
89% / 50 *

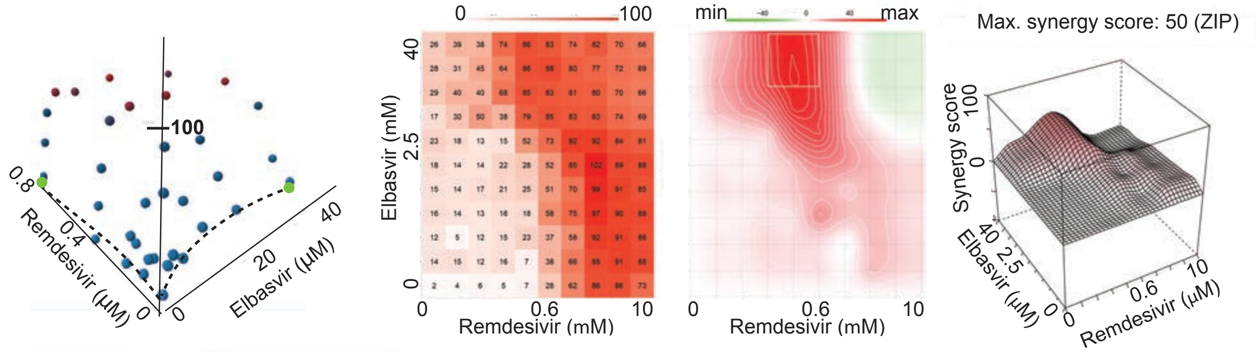

Empagliflozin/RDV
(SGLT2 Inhibitor)
82% / 40

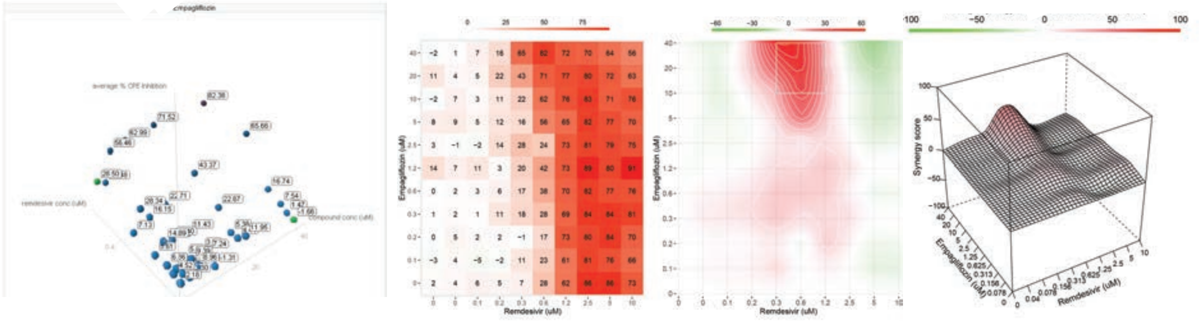

Ezetimibe/RDV
(Sterol transporter Inhibitor)
89% / 44 *

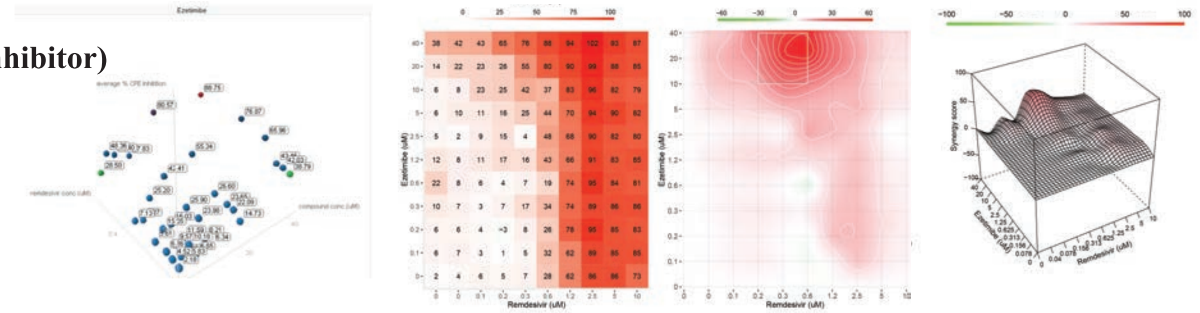

Irbesartan/RDV
(Angiotensin Receptor Blocker)
75% / 33

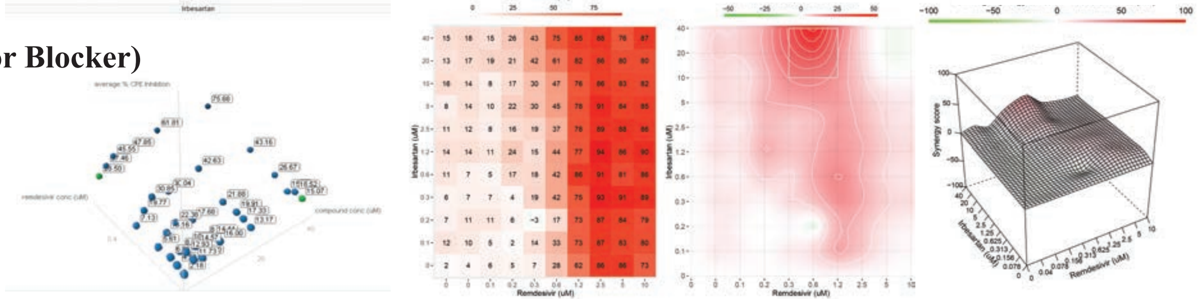

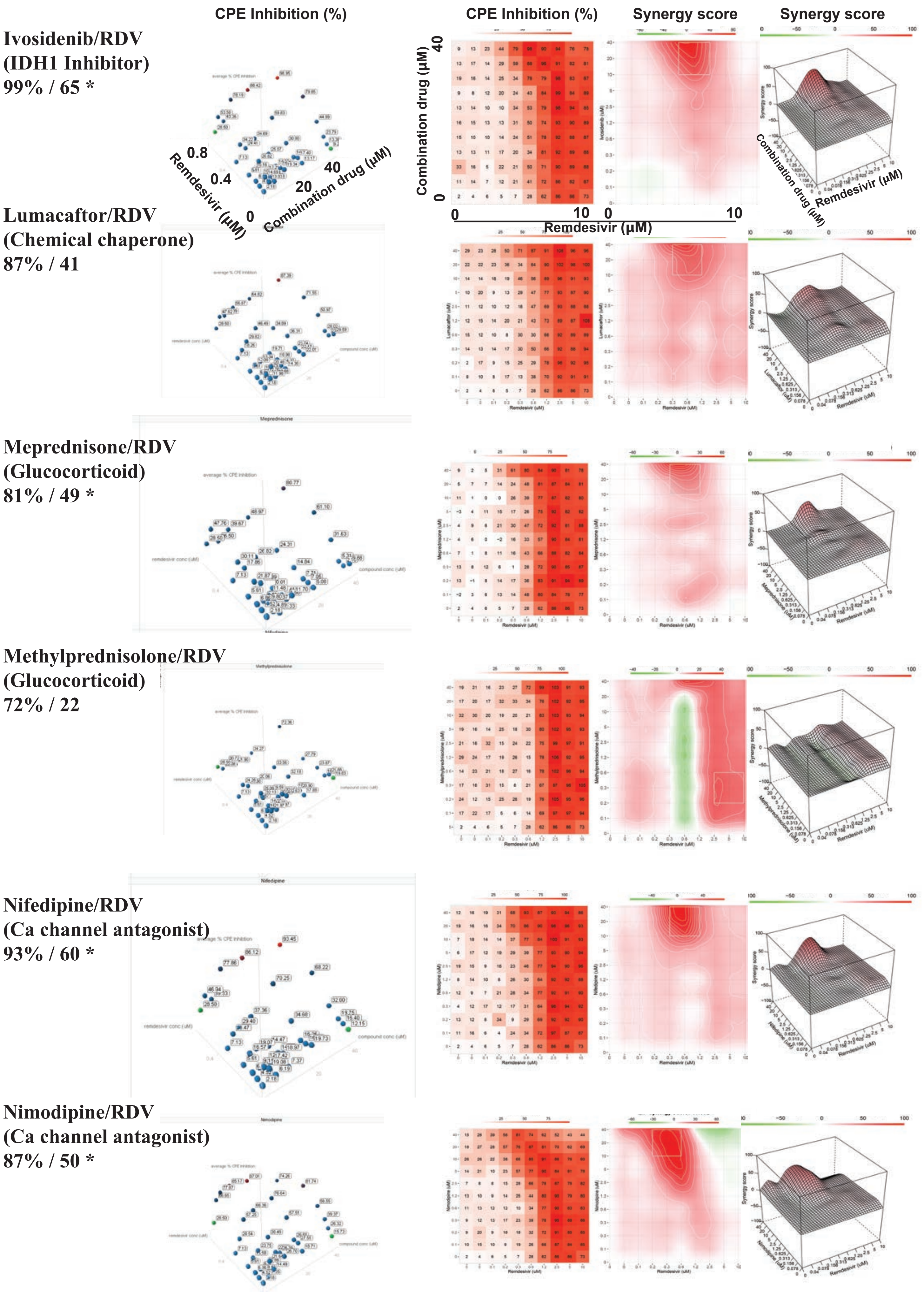

CPE Inhibition (%)
CPE Inhibition (%)
Synergy score
Synergy score
Ivosidenib/RDV
(IDH1 Inhibitor)
99% / 65 *
Remdesivir (μM)
Combination drug (μM)
Remdesivir (μM)
Lumacaftor/RDV
(Chemical chaperone)
87% / 41
Meprednisone/RDV
(Glucocorticoid)
81% / 49 *
Methylprednisolone/RDV
(Glucocorticoid)
72% / 22
Nifedipine/RDV
(Ca channel antagonist)
93% / 60 *
Nimodipine/RDV
(Ca channel antagonist)
87% / 50 *

Omeprazole sulfide/RDV
(Proton pump inh. intermed.)
95% / 44 *

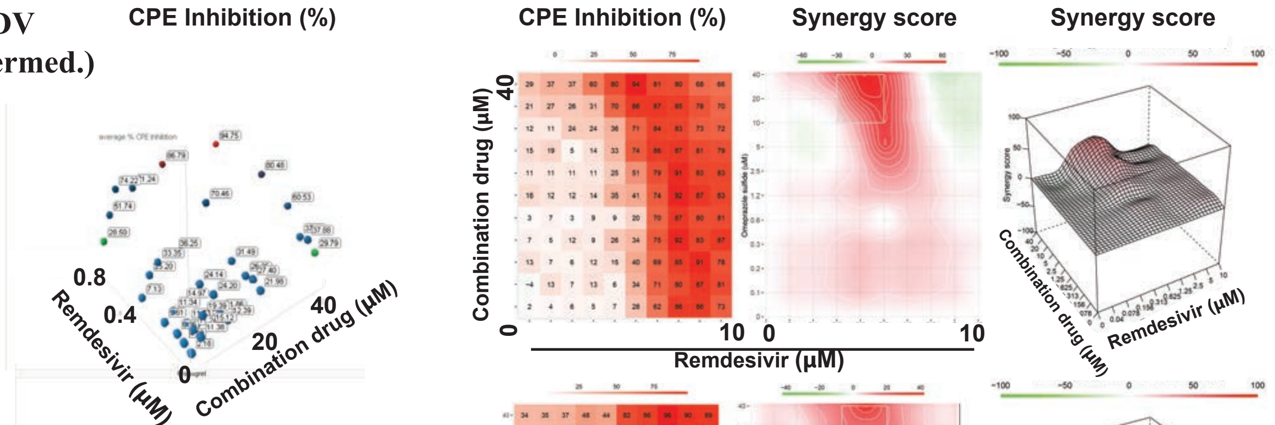

Prasugrel/RDV
(Platelet inhibitor)
83% / 28

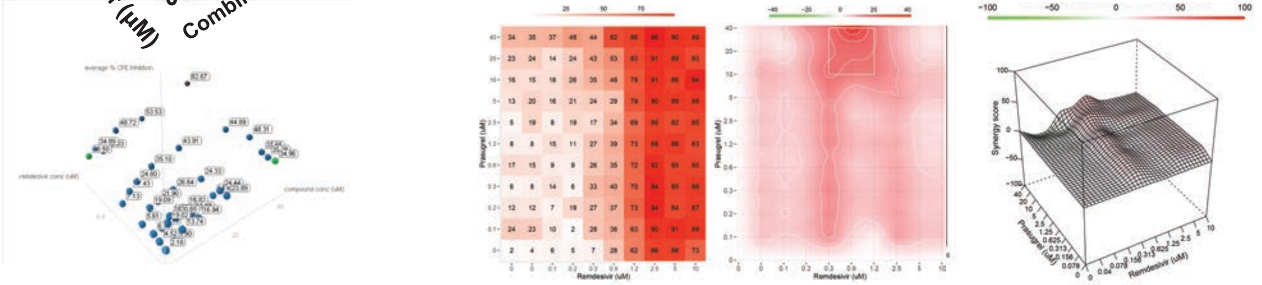

Quinapril/RDV
(ACE inhibitor)
85% / 53 *

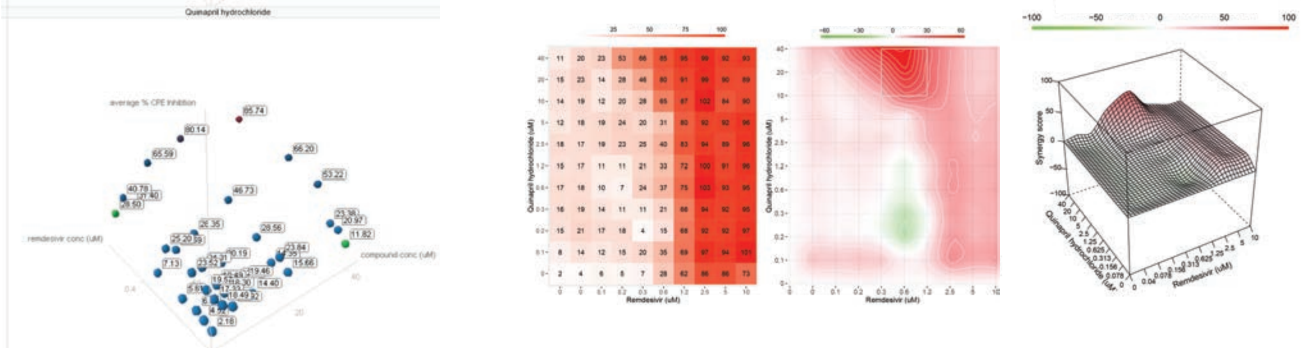

Rifamycin/RDV
(Antibiotic)
58% / 23

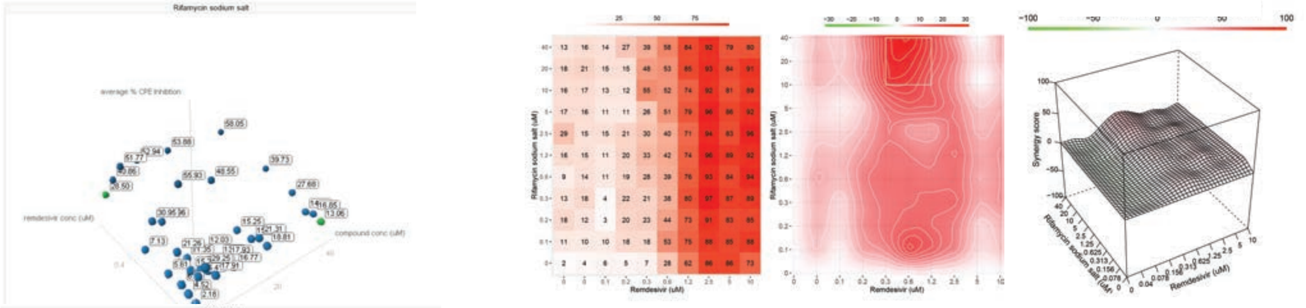

Rifaximin/RDV
(Antibiotic)
75% / 49 *

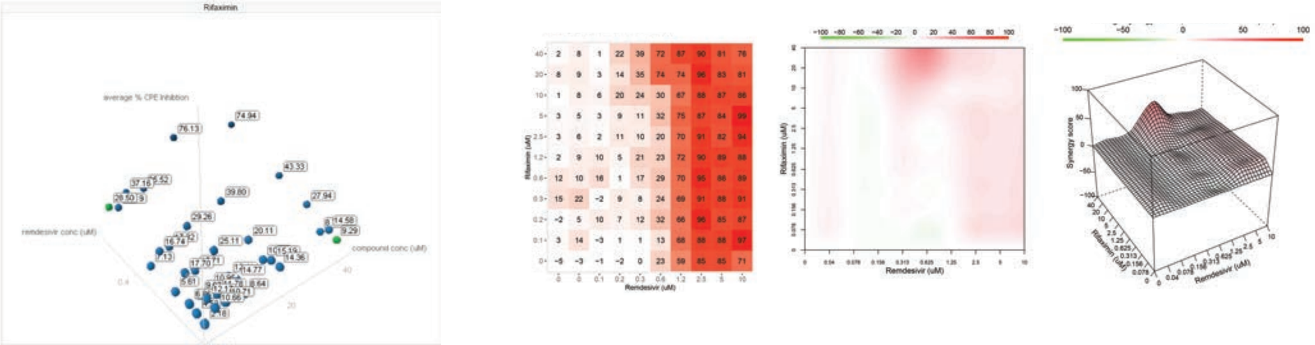

Selexipag/RDV
(Prostacyclin receptor agonist)
92% / 43 *

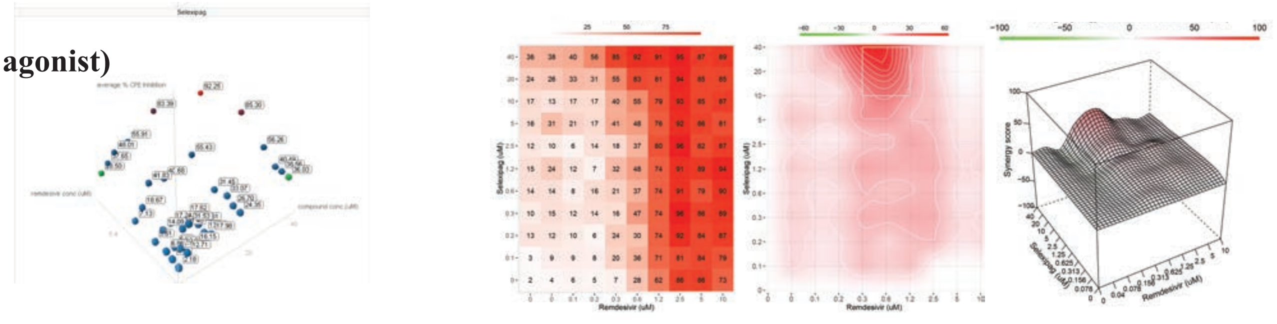

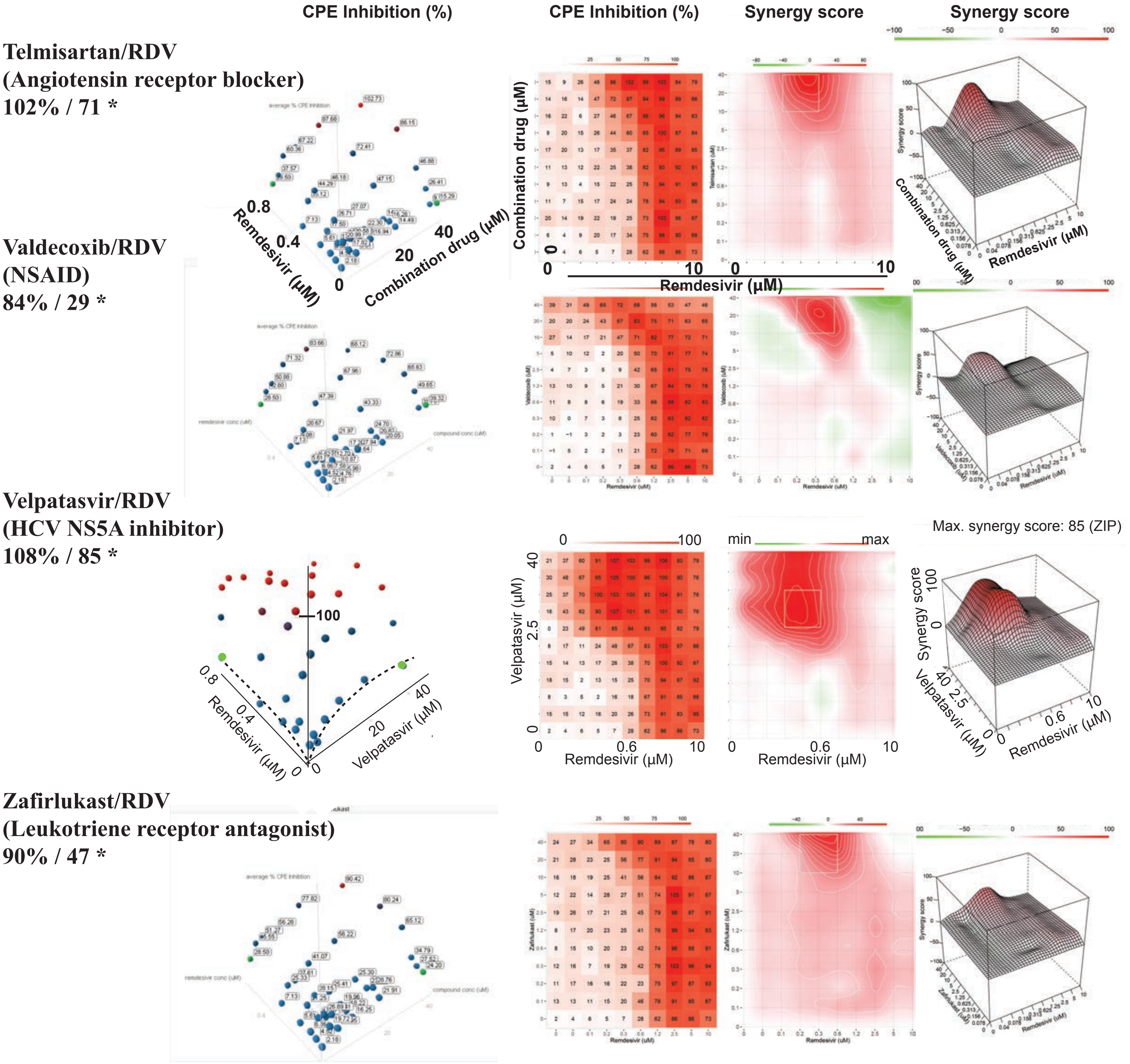

Extended Data Figure 2. Dose response interaction matrix / synergy analysis for all dose response compound combinations. Left panel: Three-dimensional plot showing synergy of combinations of labeled compound (x-axis, up to 40μM) and remdesivir (y-axis, up to 0.6μM). Z-axis indicates Inhibition of CPE (%). Markers are labeled with their respective Inhibition of CPE value; coloring gradient from blue (0% CPE inhibition) to red (100% CPE inhibition). Green – highest concentration of labeled compound and remdesivir alone. Maximum %CPE inhibition and maximum synergy score indicated under drug name. Asterisk indicates 20 prioritized compounds. **Middle left panel:** Two-dimensional representation of dose response interaction matrix. X-axis – Remdesivir (up to 10μM), y-axis: labeled compound (up to 40μM). Color gradient indicates Inhibition of CPE (%); white – 0%, red- 100%. **Middle right panel:** Topographic two-dimensional map of synergy scores determined in synergyfinder 33 from the data on the left, color gradient indicates synergy score (red – highest score). **Right panel:** Three-dimensional surface plot representing synergy score (z-axis) for each compound combination. X-axis: remdesivir up to 10μM, y-axis: labeled compound up to 40μM.

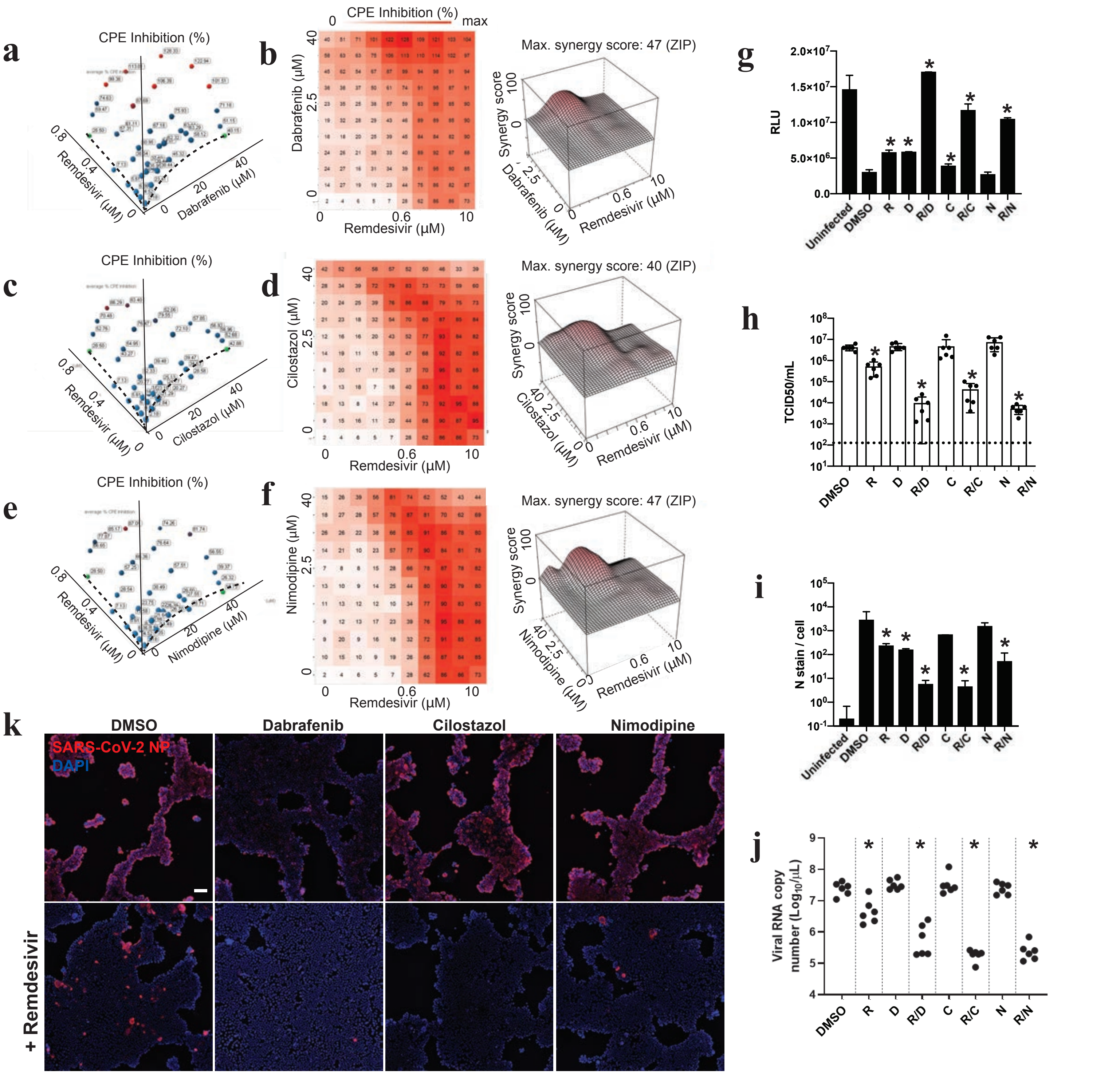

Extended Data Figure 3. Synergy of dabrafenib, cilostazol and nimodipine with remdesivir in Calu-3 cells infected with SARS-CoV-2. (a) (c) (e) Three-dimensional plot showing synergy of combinations of Dabrafenib (a), Cilostazol (c), Nimodipine (e) (x-axis, up to 40µM) and remdesivir (y-axis, up to 0.6µM). Z-axis indicates CPE Inhibition (%). Markers are labeled with their respective Inhibition of CPE value; coloring gradient from blue (0% CPE inhibition) to red (100% CPE inhibition). Green – highest concentration of tested drug and remdesivir alone, reaching ~20% Inhibition of CPE. Dashed line indicates dose response of each single agent. (b) (d) (f) Two-dimensional representation of dose response interaction matrix (left panel) and three-dimensional topographic map of synergy score (z-axis) over dose response matrix. X-axis – remdesivir (up to 10µM), y-axis: Dabrafenib (b), cilostazol (d), nimodipine (f) (up to 40µM). Color gradient indicates Inhibition of CPE (%); white – 0%, red- 100%. (g) (h) (i) (j) Confirmation of observed activities in orthogonal assays. R: remdesivir; D: dabrafenib; C: cilostazol; N: nimodipine (g) Cell-Titer Glo positive control, (h) TCID50 assay, (i) Immunofluorescence microscopy assay, quantified (j) RT-qPCR assay. Experimental conditions as in Figure 5. (k) Representative images from (i), Calu-3 cells infected with SARS-CoV-2 48hpi. Scale bar corresponds to 100µm.

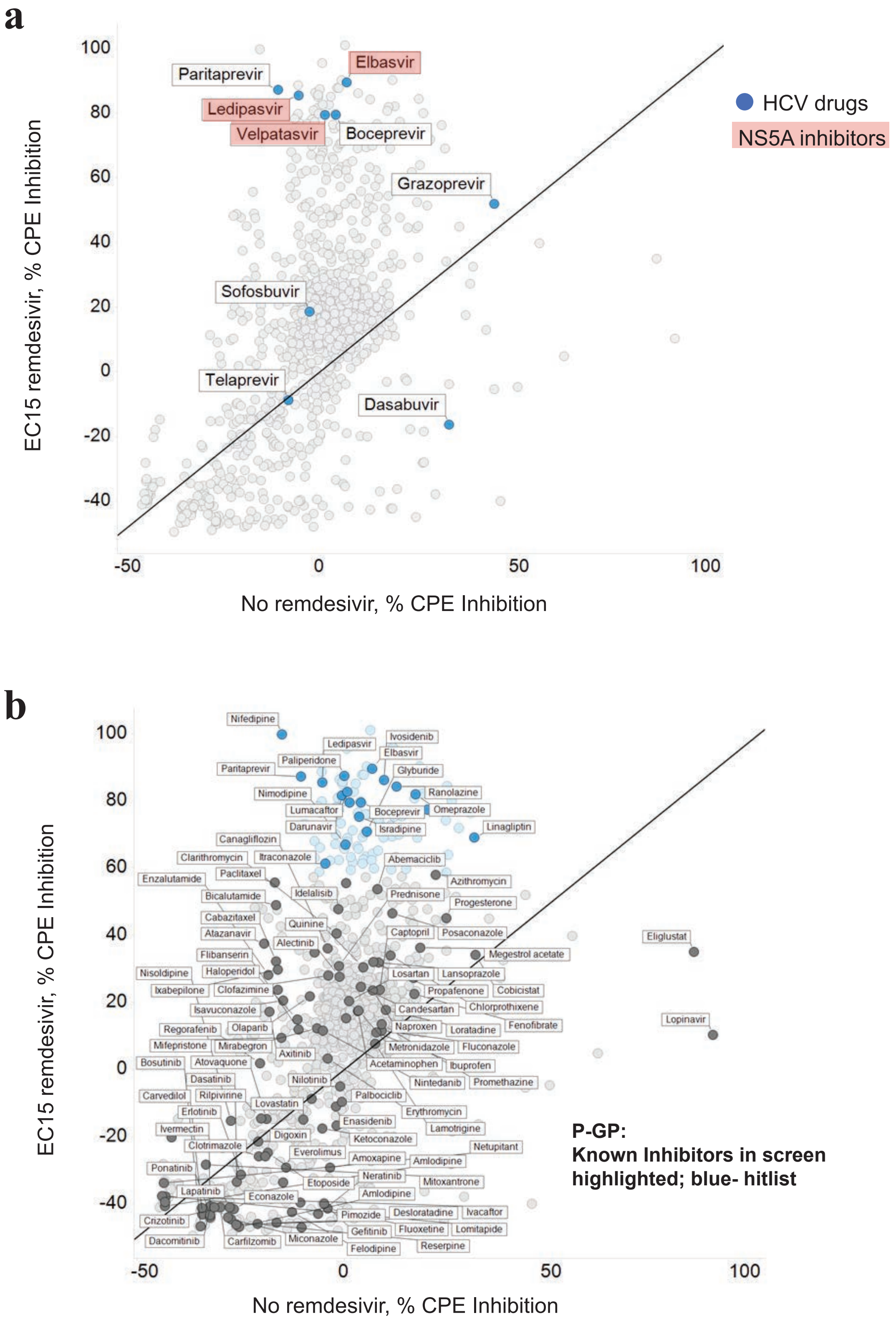

Extended Data Figure 4. Distribution of NS5A and P-gp inhibitors in the dataset (a) Screening data of all approved drugs in Vero-E6 cells infected with SARS-CoV-2, HCV drugs highlighted in blue, subset of NS5A inhibitors highlighted in red. Axes show inhibition of CPE (%), in absence (x) and presence (y) of EC15 of remdesivir. (b) as in (a), but with all known P-gp inhibitors highlighted, showing similar distribution in hitlist (blue) and inactive compounds (gray).

TABLE S1

Molecule Name	Concentration	Primary synergy screen in Vero-E6 (**Figure 1D, 1E**)				Confirmatory synergy screen in Vero-E6 (**Figure 1F**)				Synergy screen in Calu3s (**Figure 1G**)		Synergy scores in Calu3 cells (**Figure 3, Figure ED2**)	
		% CPE Inhibition with 0uM remdesivir	Cell Viability (%) with 0uM remdesivir	% CPE Inhibition with 1uM remdesivir	Cell Viability (%) with 1uM remdesivir	% CPE Inhibition with 0uM remdesivir	Cell Viability (%) with 0uM remdesivir	% CPE Inhibition with 1uM remdesivir	Cell Viability (%) with 1uM remdesivir	% CPE Inhibition with 0uM remdesivir	% CPE Inhibition with 0.15uM remdesivir	Synergy score	Most synergistic area score
Alfuzosin hydrochloride	40uM	11.6	92.9	69.5	78.4	-8.13	100.00	69.50	78.40	7.17	12.5		
Amcinonide	40uM	-5.69	59.1	79.3	91	-22.80	95.70	79.30	91.00	12.1	35		
Amprenavir	40uM	-0.525	105	84.9	101	-8.66	98.00	84.90	101.00	12	58.1		
Apalutamide	40uM	4.38	85.2	69.3	81.7	-2.05	99.60	69.30	81.70	16.5	30.5		
Betamethasone 17-valerate	40uM	-0.867	51.7	73.3	79.7	-20.90	97.10	73.30	79.70	7.26	49.1		
Bexarotene	40uM	14.2	78.5	76.7	82.5	8.38	112.00	76.70	82.50	23	75.7	**5**	**19**
Boceprevir	40uM	3.89	69.8	79.5	85.1	-12.90	104.00	79.50	85.10	14.4	39.7		
Budesonide	40uM	-5.41	66	79.8	82	-34.50	90.60	79.80	82.00	74.1	72.1	**4**	**17**
Calcifediol	40uM	5.3	107	63.4	92.4	-2.76	106.00	63.40	92.40	17.6	34.7		
Chloroquine diphosphate	40uM	24.9	87.2	63.9	76.6	71.30	101.00	63.90	76.60	10.5	8.98		
Cilostazol	40uM	4.93	61.1	70.4	71.3	-6.42	106.00	70.40	71.30	12	64.5	**10**	**36**
Cisapride	40uM	4.26	74	87.8	90.4	-25.80	103.00	87.80	90.40	7.08	20.5		
Clevidipine butyrate	40uM	2.21	91.5	82.6	86	-25.80	99.80	82.60	86.00	11.9	41.7		
Clindamycin	40uM	0.474	85	95.6	86.5	-16.30	108.00	95.60	86.50	8.38	44.8		
Clobetasol propionate	40uM	-1.64	79.7	76.2	81.4	-22.80	90.90	76.20	81.40	45	79.3	**14**	**41**
Conivaptan hydrochloride	40uM	-7.13	82.5	68	81.7	-17.30	92.80	68.00	81.70	23.4	90	**14**	**59**
Dabigatran etexilate	40uM	18.7	62.3	82.2	88.4	55.50	112.00	82.20	88.40	11.2	28.6		
Dabrafenib	40uM	25.8	67.7	80	89.8	74.40	112.00	80.00	89.80	16.5	109	**18**	**56**
Darunavir	40uM	-0.58	68.2	81.6	85.7	-15.20	108.00	81.60	85.70	15.6	58		
Deflazacort	40uM	6.83	91.4	64.3	78.3	-7.32	95.20	64.30	78.30	2.42	44.6		
Dienogest	40uM	14	87.9	62.3	76.4	4.73	103.00	62.30	76.40	4.91	27.4		
Doxapram hydrochloride monohydrate	40uM	-5.45	88	88.7	85.7	-29.60	101.00	88.70	85.70	7.05	54.7		
Droperidol	40uM	-1.14	79.1	86.3	79.9	3.66	113.00	86.30	79.90	2.44	16.8		
Drospirenone	40uM	6.31	49.9	101	87.7	-13.00	92.50	101.00	87.70	8.92	85.4	**4**	**25**
Dutasteride	40uM	-7.14	66.6	63	81.2	-18.50	95.00	63.00	81.20	21.5	36		
Dypyridamole	40uM	13.8	78.4	75.4	79.5	-9.44	104.00	75.40	79.50	9.69	65.2		
Elbasvir	40uM	6.7	101	89.5	92.1	-11.70	97.40	89.50	92.10	17.4	69.3	**13**	**49**
Empagliflozin	40uM	8.49	86.1	73.2	67.9	2.56	101.00	73.20	67.90	5.76	69.3	**4**	**28**
Erythromycin ethylsuccinate	40uM	-1.98	83.5	78	84	-14.20	104.00	78.00	84.00	13	44.5		
Esomeprazole Magnesium	40uM	18.1	63	90.2	96.9	9.35	111.00	90.20	96.90	37.4	65.9		
Ezetimibe	40uM	0.679	78.6	68.9	75.5	-9.40	101.00	68.90	75.50	13.8	67.8	**8**	**31**
Farlutin	40uM	3.08	72.3	66.5	71	-6.72	95.10	66.50	71.00	18.2	17.6		
Fidaxomicin	40uM	4.63	67.9	87.8	86.4	17.10	99.90	87.80	86.40	13.7	54.7		
Finasteride	40uM	11.1	85.2	72.6	72	-9.08	92.90	72.60	72.00	10.4	6.3		
Fulvestrant	40uM	-1.68	83.5	80.8	84	-19.50	105.00	80.80	84.00	0.341	37.4		
Glyburide	40uM	5.42	62.1	70.8	76.3	-10.60	99.80	70.80	76.30	4.55	33.8		
Hydroxychloroquine sulfate	40uM	17.6	49.4	69.1	97.9	35.40	104.00	69.10	97.90	6.32	19.3		
Imiquimod	40uM	-2.1	60.2	69.9	81.4	-8.31	105.00	69.90	81.40	6.18	29.5		
Indacaterol maleate	40uM	4.5	55.5	62.6	71.4	-17.80	97.20	62.60	71.40	4.4	2.31		
Irbesartan	40uM	12.1	89.9	71.6	88	-9.93	98.00	71.60	88.00	12.6	82.3	**7**	**24**
Isradipine	40uM	3.52	49.5	75.3	78.5	-31.10	97.60	75.30	78.50	14.7	30.6		
Ivabradine hydrochloride	40uM	0.608	57.2	67.9	91.2	-21.40	103.00	67.90	91.20	5.01	29.3		
Ivosidenib	40uM	9.55	90.4	86.1	85.8	17.20	100.00	86.10	85.80	4.48	98.4	**11**	**45**
Ledipasvir	40uM	-5.28	103	85.5	86.4	-41.00	96.80	85.50	86.40	23.8	68.9		
Lenvatinib	40uM	-5.85	81.7	66.1	79.3	-22.20	99.90	66.10	79.30	15.4	21.1		
Linagliptin	40uM	31.3	82.3	69	78.4	16.70	105.00	69.00	78.40	0.757	22.3		
Loteprednol etabonate	40uM	2.02	78.1	71.2	82.9	-41.60	100.00	71.20	82.90	6.64	27.8		
Luliconazole	40uM	11.9	91.1	75.3	94.7	-6.93	102.00	75.30	94.70	16.9	31.9		
Lumacaftor	40uM	0.673	94.2	82.6	94.7	-4.63	104.00	82.60	94.70	2.1	86.8	**12**	**29**
Lurasidone hydrochloride	40uM	-11	67.8	71.7	81.9	-22.50	101.00	71.70	81.90	9.38	21.6		
Macitentan	40uM	-19.4	66.2	82.2	105	-30.70	109.00	82.20	105.00	4.53	39.3		
Medrysone	40uM	-5.82	45.7	76.5	83.5	-15.70	100.00	76.50	83.50	13.5	27.5		
Meprednisone	40uM	-5.08	77.8	76.8	85.1	-28.80	98.90	76.80	85.10	5.81	77.5	**7**	**25**
Methylprednisolone	40uM	3.4	86.5	65.8	82.9	-3.04	99.50	65.80	82.90	1.32	88.9	**7**	**17**
Nandrolone decanoate	40uM	5.45	88	90.3	78.5	-10.70	102.00	90.30	78.50	16.3	29.1		
Nifedipine	40uM	-15	40.4	99.8	92.4	-58.10	71.00	99.80	92.40	11.7	80.3	**12**	**43**
Nimodipine	40uM	0.237	77.2	66.9	74.6	-22.50	102.00	66.90	74.60	19.8	70.5	**10**	**43**
Nizatidine	40uM	3.28	57.7	76.4	82.3	-13.40	103.00	76.40	82.30	8.59	25.1		
Omeprazole	40uM	17.1	83.9	81.9	90.6	-3.03	106.00	81.90	90.60	16.4	30.7		
Omeprazole sulfide	40uM	16.8	89.8	80.7	89	2.28	99.00	80.70	89.00	15.4	77.1	**7**	**32**
Ondansetron hydrochloride	40uM	24.7	104	84.5	92	20.00	96.40	84.50	92.00	7.3	10.1		
Paliperidone	40uM	-0.104	57.1	87.4	82.6	-12.60	97.80	87.40	82.60	10.6	20.3		
Paritaprevir	40uM	-10.5	61.2	87.2	94.6	3.56	109.00	87.20	94.60	9.65	20.4		
Pazopanib	40uM	11.7	65.5	95.8	90.9	-6.84	102.00	95.80	90.90	2.37	29.9		
Permethrin	40uM	30.4	76.6	76.3	85.4	17.10	103.00	76.30	85.40	27.2	57.4		
Phenoxybenzamine hydrochloride	40uM	-1.32	48.3	62.4	80.6	-17.60	102.00	62.40	80.60	8.46	22.2		
Phenylbutazone	40uM	7.76	87.3	76.5	88.8	-6.46	110.00	76.50	88.80	7.29	69.6		
Prasugrel	40uM	-0.407	51.1	85.7	95.2	-3.18	112.00	85.70	95.20	3.31	57.2	**8**	**17**
Quinapril hydrochloride	40uM	2.71	66.2	85.2	87.8	-3.03	116.00	85.20	87.80	9.11	88.6	**10**	**35**
Quinidine hydrochloride	40uM	27.6	83.5	71.9	88.8	27.20	100.00	71.90	88.80	9.25	23.6		
Ranolazine	40uM	12.6	109	84.3	90.7	-19.10	105.00	84.30	90.70	9.99	19.2		
Ranolazine dihydrochloride	40uM	-3.12	54.5	76.6	88.4	-26.10	104.00	76.60	88.40	9.44	19.1		
Rebeprazole sodium	40uM	1.58	59.8	63.2	66.8	-13.30	93.80	63.20	66.80	28.8	30.1		
Rifamycin sodium	40uM	8.07	89.5	65.1	71.1	-2.41	100.00	65.10	71.10	4.08	93.5	**8**	**20**
Risperidal	40uM	-2.2	82	67.2	92.7	-26.10	98.00	67.20	92.70	8.29	28.4		
Selexipag	40uM	9.43	62.1	69	71.7	-12.40	104.00	69.00	71.70	31.4	100	**9**	**29**
Sildenafil citrate	40uM	5.81	90.7	76	80.8	-32.40	90.10	76.00	80.80	1.98	26.2		
Silodosin	40uM	0.0161	93	65.7	84.9	-12.70	89.70	65.70	84.90	5.51	16.5		
Tacrine hydrochloride hydrate	40uM	6.53	85.4	62.3	84.4	33.10	94.90	62.30	84.40	5.74	19.8		
Tamsulosin hydrochloride	40uM	5.14	61.9	75.9	90.8	4.87	102.00	75.90	90.80	9.37	30.1		
Telmisartan	40uM	19.9	63.3	77.3	84.7	4.71	102.00	77.30	84.70	15.5	94.5	**13**	**48**
Tetrabenazine	40uM	8.18	103	74.4	85.7	-5.75	102.00	74.40	85.70	19	29.8		
Tetrabenazine (racemate)	40uM	12.4	75.6	63.3	71.9	-9.28	102.00	63.30	71.90	14.1	40.6		
Tyramine	40uM	-0.338	68.6	79.9	90.4	0.79	100.00	79.90	90.40	10.4	48.9		
Ulipristal acetate	40uM	-3.47	64.7	62.1	61.8	-13.70	102.00	62.10	61.80	5.08	24.3		
Valdecoxib	40uM	3.77	85.8	79.9	82.4	-11.10	98.20	79.90	82.40	17.5	78.5	**1**	**25**
Vardenafil dihydrochloride	40uM	24.4	104	74.1	83.9	-4.02	93.80	74.10	83.90	5.35	61.5		
Velpatasvir	40uM	1.17	72.8	79.5	89.5	-20.50	109.00	79.50	89.50	40.9	88.3	**23**	**74**
Vismodegib	40uM	-1.35	82.5	77.4	83.2	-13.20	97.30	77.40	83.20	4.56	49.1		
VX661	40uM	-6.28	77.3	78.3	75.1	-24.90	101.00	78.30	75.10	10.5	14.3		
Zafirlukast	40uM	13.1	106	69.9	80.8	-2.61	115.00	69.90	80.80	21.4	94.8	**13**	**35**
